# Cellular Origins and Pathogenesis of Gastrointestinal NK- and T-Cell Lymphoproliferative Disorders

**DOI:** 10.3390/cancers14102483

**Published:** 2022-05-18

**Authors:** Susan Swee-Shan Hue, Siok-Bian Ng, Shi Wang, Soo-Yong Tan

**Affiliations:** 1 Department of Pathology, National University Hospital, Singapore 119074, Singapore; swee_shan_hue@nuhs.edu.sg (S.S.-S.H.); shi_wang@nuhs.edu.sg (S.W.); 2 Department of Pathology, Yong Loo Lin School of Medicine, National University of Singapore, Singapore 119074, Singapore; patnsb@nus.edu.sg; 3 Cancer Science Institute of Singapore, National University of Singapore, Singapore 117599, Singapore

**Keywords:** CD8αα+ T-cells, iCD3+ ILC, iCD8α ILC, NK cells, ENKTL, Indolent T-cell LPD, NK-cell enteropathy, lymphomatoid gastropathy, EATL, MEITL, ITCL, NOS

## Abstract

**Simple Summary:**

Intestinal T- and NK-cell lymphoproliferative disorders are a group of rare gastrointestinal disorders that arise from immune cells in the intestinal mucosa that are also relatively unknown. Diseases such as indolent T-cell lymphoproliferative disorders of the gastrointestinal tract do not even require treatment, whereas others, such as monomorphic epitheliotropic intestinal T-cell lymphoma, will generally cause death within a year. No effective treatment is currently available, as little is known about how these tumours form or even what cells they arise from. This article summarizes the current state of knowledge about the main types of immune cells in the gastrointestinal mucosa and the processes by which they may transform into neoplasms. The clinical behaviour, pathological appearances and the molecular alterations that underlie these diseases are also discussed.

**Abstract:**

The intestinal immune system, which must ensure appropriate immune responses to both pathogens and commensal microflora, comprises innate lymphoid cells and various T-cell subsets, including intra-epithelial lymphocytes (IELs). An example of innate lymphoid cells is natural killer cells, which may be classified into tissue-resident, CD56^bright^ NK-cells that serve a regulatory function and more mature, circulating CD56^dim^ NK-cells with effector cytolytic properties. CD56^bright^ NK-cells in the gastrointestinal tract give rise to indolent NK-cell enteropathy and lymphomatoid gastropathy, as well as the aggressive extranodal NK/T cell lymphoma, the latter following activation by EBV infection and neoplastic transformation. Conventional CD4+ TCRαβ+ and CD8αβ+ TCRαβ+ T-cells are located in the lamina propria and the intraepithelial compartment of intestinal mucosa as type ‘a’ IELs. They are the putative cells of origin for CD4+ and CD8+ indolent T-cell lymphoproliferative disorders of the gastrointestinal tract and intestinal T-cell lymphoma, NOS. In addition to such conventional T-cells, there are non-conventional T-cells in the intra-epithelial compartment that express CD8αα and innate lymphoid cells that lack TCRs. The central feature of type ‘b’ IELs is the expression of CD8αα homodimers, seen in monomorphic epitheliotropic intestinal T-cell lymphoma (MEITL), which primarily arises from both CD8αα+ TCRαβ+ and CD8αα+ TCRγδ+ IELs. EATL is the other epitheliotropic T-cell lymphoma in the GI tract, a subset of which arises from the expansion and reprograming of intracytoplasmic CD3+ innate lymphoid cells, driven by IL15 and mutations of the *JAK-STAT* pathway.

## 1. Introduction

Various subsets of natural killer (NK) and T-cell populations exist in the normal gastrointestinal (GI) mucosa, with distinct developmental pathways, phenotypes and functions. Lymphoproliferative disorders (LPDs) of NK- and T-cell lineages that arise from these cell populations can be better understood from the perspective of the normal NK- and T-cell counterparts in GI mucosa.

NK- and T-cell lymphomas of the GI tract are uncommon, comprising 8–15% of all GI lymphomas [1,2]. They include monomorphic epitheliotropic intestinal T-cell lymphoma (MEITL), enteropathy-associated T-cell lymphoma (EATL), intestinal T-cell lymphoma, not otherwise specified (ITCL, NOS), indolent T-cell LPD of the GI tract and NK-cell enteropathy/lymphomatoid gastropathy. Other lymphomas may occasionally involve the gastrointestinal tract, but only extranodal NK/T-cell lymphoma (ENKTL) will be considered in this review, as it occurs with some frequency, particularly in Asia.

## 2. Immune Cells in the Gastrointestinal Mucosa

The innate arm of the immune system is the first line of defence against invading pathogens, which are recognized by the pattern recognition receptors and Toll-like receptors expressed in macrophages, neutrophils and dendritic cells. At the same time, gastrointestinal immune cells have an immunoregulatory role in limiting inflammatory responses to commensal organisms and dietary antigens, ensuring intestinal epithelial integrity and immune homeostasis.

The intestinal immune system comprises T-cells, B-cells, plasma cells, dendritic cells and a group of innate lymphoid cells that are variously located in mesenteric lymph nodes and intestinal mucosa within Peyer’s patches, cryptopatches and isolated lymphoid follicles, are diffusely distributed in the lamina propria, or as intra-epithelial lymphocytes (IELs). The latter may be subdivided into CD4+ TCRαβ+ and CD8αβ+ TCRαβ+ (induced or ‘type a’) IELs, CD8αα+ TCRαβ+ and CD8αα+ TCRγδ+ (natural or ‘type b’) IELs and TCR− innate lymphoid cell-like IELs, a subset of which displays intracytoplasmic CD3 expression (iCD3+ innate IELs). In this review, we will focus on innate lymphoid cells (particularly NK cells) and T-cells in the gastrointestinal immune system, as well as the lymphoproliferative disorders that may arise from them.

### 2.1. Innate Lymphoid Cells (ILCs)

Innate lymphoid cells (ILCs) such as natural killer (NK) cells in the gastrointestinal tract are important effectors of the innate immune system with a central role in the early phase of the immune response, in tissue repair and remodelling, containment of commensal microbes and maintenance of epithelial cell integrity. Located in the lamina propria and between epithelial cells, they are defined by the lack of antigen-specific receptors, lack of myeloid and dendritic cell markers and having lymphocyte morphology.

#### 2.1.1. Lamina Propria ILCs

ILCs may be divided into three broad categories based on expression of Th1-type cytokine (IFNγ) in group 1, Th2-type cytokines (IL5, IL13) in group 2 and Th17-type cytokines (IL17A, IL22) in group 3 ILCs [3,4,5]. The Id2 (inhibition of DNA binding) pathway is central to the development of all groups of ILCs except iCD3+ ILCs.

NK cells and other Group 1 ILCs (ILC1) express IFNγ [3]. NK cells express T-bet and Eomesodermin (Eomes) [6] whilst NK-like ILC1 cells express T-bet but with reduced Eomes expression [7,8,9]. ILC1s are rare in the normal intestinal mucosa but are enriched in patients with Crohn’s disease [10,11]. Group 2 ILCs (ILC2) produce Th2-type cytokines such as IL5, IL13, express GATA3 and RORα [12], and have a key role in defence against helminthic infections [3,13,14,15]. The third group of ILCs (ILC3) produces Th17-type cytokines (such as IL17A and IL22) and expresses aryl hydrocarbon receptor (AhR) and retinoic acid receptor orphan receptor gamma (RORγt) [16,17,18]. They are located in the small and large intestines. ILC3s may be subdivided into natural cytotoxicity receptor (NCR)+ ILC3s, NCR− ILC3s and lymphoid tissue inducer (LTi) cells [9], the latter with a key role in the formation of secondary lymphoid organs during embryogenesis [19]. Apart from regulatory ILCs (which are the innate cell counterparts of regulatory T-cells) [20], the five types of ILCs (namely, NK cells, ILC1, ILC2, ILC3 of both NCR+ and NCR- subsets and LTi cells) [9] may be separated based on immunophenotype, types of cytokines secreted and differences in developmental pathways and functions (Table 1).

#### 2.1.2. Intraepithelial Innate Lymphoid Cells (ILCs)

Although most ILCs are located in the lamina propria, ILCs are also present in the intraepithelial compartment as non-T-cell IELs. These include:

(a) Intraepithelial ILC1 (IE-ILC1) cells that are Id2-dependent, secrete IFN-γ and express T-bet, NKp44, CD103 but not CD127 [11,22,23];
(b) A minor subset that resembles intraepithelial ILC3 (IE-ILC3) with expression of NKp44, CD103 and production of IL22 [23,24];
(c) Intraepithelial ILCs that are Id2-independent and express intracytoplasmic CD3 as innate CD3+ IEL (iCD3+ IEL) [25];
(d) ILCs that express both intracytoplasmic CD3 as well as CD8αα homodimers, referred to as innate CD8αα+ IELs (iCD8α IEL) [25,26,27].

Both iCD3+ and iCD8α IELs are dependent on IL15 but differentiate via an Id2-independent pathway, unlike other ILCs. They secrete IFN-γ, express T-bet as well but with low to absent Eomes expression.

### 2.2. Natural Killer (NK) Cells

NK-cells, which are traditionally defined by their CD3− CD56+ phenotype, display cytolytic activity and are important in the elimination of viruses and tumour cells. Whilst NK-cells do not express T-cell receptors (TCR) and surface CD3 (sCD3), activated NK-cells may upregulate cytoplasmic CD3ε [28], which is the target of most CD3 antibodies for immunohistochemistry in formalin-fixed paraffin-embedded (FFPE) material. NK-cells may be divided into a CD56^dim^ CD16+ subset that demonstrates effector cytotoxic function and a CD56^bright^ CD16+/− subset that is weakly cytotoxic, produces cytokines and displays a regulatory function.

#### 2.2.1. CD56^dim^ versus CD56^bright^ NK-Cells

CD56^dim^ NK-cells are mainly located in peripheral blood, and they express CD16, CD122, CD158/KIR (killer immunoglobulin-like receptors), with the most mature forms expressing CD57 [29] but not CD25 nor CD117 unless activated. Conversely, CD56^bright^ CD16−KIR− NK-cells may co-express CD25 and CD117, are primarily resident in tissues (marrow, spleen, lymph node) and are considered the precursors to more mature CD56^dim^ CD16+ KIR+/− NK-cells [30,31,32]. In addition, CD56^bright^ NK-cells express CD11c, CD62L/L-selectin, CD94/NKG2 and CD335/NKp46 [33,34] (Table 2). CD336/NKp44 is expressed on CD56^bright^ NK cells when activated by pro-inflammatory cytokines IL2, IL15 and IL1β [35,36]. CD56^dim^ NK cells display cytolytic activity whilst the CD56^bright^ subset is a potent producer of inflammatory cytokines [37,38] following activation by monokines such as IL12, IL15 and IL18.

There is some degree of plasticity between CD56^bright^ and CD56^dim^ populations of NK cells. During inflammation and following activation by IL2, CD56^bright^ NK cells express CD158/KIR, CD16, perforin and acquire cytolytic properties similar to CD56^dim^ NK cells [32,39]. Conversely, when exposed to IL12, CD56^dim^ NK cells upregulate CD56 and downregulate CD16 [40].

#### 2.2.2. CD4 and CD8 Expression in NK-Cells

Although most NK cells are CD4− CD8−, CD8αα homodimers may be expressed in NK cells, as they do in subsets of T-cells. CD8αα enhances the interaction of MHC class I molecules with KIR3DL1, an inhibitory receptor of NK cells, leading to inhibition of NK-cell activation [41]. NK-cells resident in lymphoid tissue also express higher levels of CD4 compared to those in peripheral blood [42]. CD4 in activated NK-cells acts as a chemokine receptor and increases cytokine production.

#### 2.2.3. Tissue-Resident NK Cells

In contrast to CD56^dim^ NK cells in peripheral blood, tissue-resident CD56^bright^ NK-cells are CD16- and commonly express CD69 [43], chemokine receptors (CXCR6, CCR5) [43,44,45], and some adhesion molecules (e.g., CD49a) [45], but not others (e.g., CD62L) [46]. Together, these molecules prevent egress of NK-cells from the tissue site.

#### 2.2.4. NK Cells in the GI Tract

The role of NK-cells in the human GI tract has been reviewed by Poggi et al. [47]. In keeping with CD56^bright^ NK-cells, they do not express CD16 and lack strong cytolytic activity. They are mainly located in the intestinal epithelium as intraepithelial lymphocytes (IELs) but may be seen in the lamina propria and express IFNγ in response to IL12 [47,48]. NK-cells express T-bet and produce Th1-like cytokines such as IFNγ and TNFα [49] during inflammatory responses.

## 3. T-Cell Populations in the Gastrointestinal Tract

T-cells within intestinal mucosa comprise conventional CD4+ helper and CD8+ cytotoxic T-cells that primarily populate the lamina propria, as well as unconventional T-cells mainly located in the intraepithelial compartment that display regulatory rather than effector properties.

### 3.1. Conventional T-Lymphocytes

Conventional T-cells derive from developing thymocytes that have undergone positive and negative selection, migrate into gut-associated lymphoid tissue (GALT), e.g., Peyer’s patches, where they undergo antigen-driven maturation to become effector memory cells that home to intestinal mucosa. Conventional T-cells are TCRαβ+ and express CD4 or CD8αβ, which serve as co-receptors for TCR. Responding to environmental cues, conventional CD4+ T-cells may differentiate into Th1, Th2, Th17 and induced regulatory T (iTreg) cells. Conventional T-cells comprise the bulk of the T-cell population in the intestinal mucosa and may be located both in the intraepithelial compartment and in the lamina propria [50].

### 3.2. Unconventional T-Lymphocytes

Instead of proceeding to positive selection, CD4+ CD8+ thymocytes undergo agonist selection, downregulate CD4 and CD8, and develop into self-reactive precursors of CD8αα T-cells that escape negative selection [51,52]. Unconventional T-cells derived from such double-negative thymocytes express CD8αα, along with TCRγδ or TCRαβ and home directly to the gastrointestinal tract [50,53,54]. Such unconventional T-cells include CD8αα+ TCRαβ+ and CD8αα+ TCRγδ+ subsets. CD8αα acts as a TCR repressor, negatively regulates T-cell activation and assumes a regulatory role.

## 4. Intraepithelial Lymphocytes (IELs)

IELs comprise innate lymphoid cells and T-cells within the gut epithelium and can be divided into those that express TCR and a group of ILCs that lacks TCR expression [54,55,56]. TCR+ IELs can be further divided into conventional CD4+ and CD8αβ+ T-cells (type ‘a’/induced TCR+ IELs) and unconventional CD8αα+ T-cells (type ‘b’/natural TCR+ IELs) [54,55,57,58]. Intraepithelial ILCs and CD8αα+ unconventional T-cells rely on T-bet for development and exhibit expression of CD69 [8,59] and IL15 receptors [55,56].

### 4.1. Type ‘a’ (Induced TCR + IELs)

Conventional T-cells comprise the first group of IELs with CD4+ TCRαβ+ (10–15%) and CD8αβ+ TCRαβ+ phenotypes (70–80%) [58]. As with conventional T-cells, they are CD2+ CD5+ CD28+ LFA1+. CD4+TCRαβ+ IELs behave as helper T-cells whilst CD8αβ+ T-cells mediate effector cytotoxic function.

However, CD4+ TCRαβ+ T-cells may also express CD8αα upon entering the intestinal epithelial compartment [60,61]. The development of CD4+ TCRαβ+ T-cells into CD4+ CD8αα+ TCRαβ+ IELs depends on upregulation of ThPOK, reciprocal downregulation of RUNX family transcription factor 3 [62,63], expression of T-bet and aryl hydrocarbon receptor (AhR) [61,64,65].

CD8αβ+ TCRαβ+ circulating T-cells are different from IELs with the same phenotype. The latter’s development may be influenced by the high levels of TGFβ in the gut microenvironment, which induces expression of CD103 and CD8αα. Compared with circulating CD8αβ+ TCRαβ+ T-cells, CD8αβ+ TCRαβ+ IELs express granzyme B, CD103, CD69 and produce lower levels of IFNγ and TNFα [66].

### 4.2. Type ‘b’ (Natural TCR + IEL)

The second group of IELs comprises unconventional T-cells with CD8αα+ TCRαβ+ (<1%) and CD8αα+ TCRγδ+ phenotypes (5–20%) [58]. Although these IELs are thymic derived, they undergo agonist selection and are self-reactive [51]. Differentiation into type ‘b’ IELs and expression of CD8αα occur in the intestinal environment, under the influence of TGFβ and T-bet [8,67]. The Vitamin D receptor AhR is also important for their maintenance and survival [68,69].

Unlike CD4 and CD8αβ, CD8αα does not function as a TCR co-receptor and instead suppresses TCR function. These IELs lack expression of CD2, CD5, CD28 and LFA1 but instead express NKG2D (in TCRαβ+ cells) [55,70] or NKG2A (in TCRγδ+ cells) [71], CD244, Ly49 members and produce inhibitory cytokines such as IL10 and TGFβ to mediate immune tolerance [54,56,57]. TCRγδ+ T-cells may be divided into Vδ1 and Vδ2 subsets based on the TCRδ chain repertoire and the predominant subset in intestinal mucosa is Vδ1+ cells that constitutively express CD335/NKp46 [72].

### 4.3. Innate Lymphoid Cells (ILCs)

Finally, ILCs that rearrange but do not express TCR genes form the third group of IELs, which may be prominent in ileal grafts. They show expression of CD103, CD56, RORγt, AhR [23,25], NKp46, NKp44 and a subset that lacks surface CD3 but expresses cytoplasmic CD3ε and CD3γ [25,54,57]. The latter is referred to as innate CD3+ (iCD3+) IELs and may be either CD8αα+ or CD8αα−. Whilst the development of ILCs depend on Id2, iCD3+ IELs arise from Notch1-activated common lymphoid precursors under the influence of IL15. Granzyme B induced by IL15 cleaves Notch1 into an inactive peptide, thereby silencing downstream target genes that are essential for T-cell differentiation [25]. They differentiate in the absence of Id2 but require Notch1, TL and IL15 signals for development [27].

### 4.4. CD8αα Expression in IELs

Expression of CD8αα is seen in the majority of IELs and is a distinctive feature of CD8αα+TCRαβ+ and CD8αα+ TCRγδ+ (‘type b’) natural TCR+ IELs and in iCD8α+ innate IELs. A subset of CD4+ TCRαβ+ and CD8αβ+ TCRαβ+ (‘type a’)-induced TCR+ IELs may also co-express CD8αα.

The CD8 molecule exists as a dimer of two isoforms, CD8α and CD8β. Conventional CD8+ T-cells express CD8αβ, which acts as a co-receptor for TCR and is expressed on MHC class I-restricted T-cells. In contrast, CD8αα decreases antigenic sensitivity of the TCR and when co-expressed with CD8αβ, CD8αα downmodulates the functional avidity of the CD8αβ-TCR:Ag-MHC activation complex [73,74]. Transient or permanent expression of CD8αα homodimers in T-cells can be induced, regardless of MHC restriction or the presence of CD4 or CD8αβ, and may be seen in all groups of IELs [75].

## 5. Lymphomas and Lymphoproliferative Disorders Derived from NK-Cells

NK-cell is the putative cell of origin for indolent lymphomatoid gastropathy/NK-cell enteropathy and aggressive NK-cell leukaemia. Both NK-cells and CD56+ T-cells may give rise to extranodal NK/T cell lymphoma and chronic active EBV infection. Apart from aggressive NK-cell leukaemia, the other entities may involve the gastrointestinal tract.

### 5.1. Lymphomatoid Gastropathy (LG)/NK-Cell Enteropathy (NKCE)

#### 5.1.1. Incidence and Prevalence

This is a rare disease, and only 36 patients with either LG or NKCE have been reported as of 2019 [76]. Figures on global incidence are not available, and lymphomatoid gastropathy was initially recognized based on a review of only 10 cases of CD56+ atypical lymphoid infiltrates in the stomach between 1998 and 2009 [77].

#### 5.1.2. Clinical Features

This is a benign, localized lymphoproliferative disorder of NK-cells in the gastrointestinal tract. Many Japanese cases were reported in the stomach (median age: 56, M = F) [77,78,79,80], but this may relate to a national surveillance program for gastric cancer detection in the country. They tend to present incidentally and regress spontaneously with a relapse rate of 38%.

Similar cases have also been reported in Korea and the United States, being more common in the intestines and among younger patients (median age: 46, M < F). These cases are more likely symptomatic, presenting with dyspepsia, vomiting, diarrhoea, constipation, rectal bleed and loss of weight [81,82,83,84]. NK-cell gastropathy tends to regress without therapy [77,78,79,80] whilst intestinal cases may persist [81,82,83,84].

#### 5.1.3. Pathological Features

**Figure 1:**
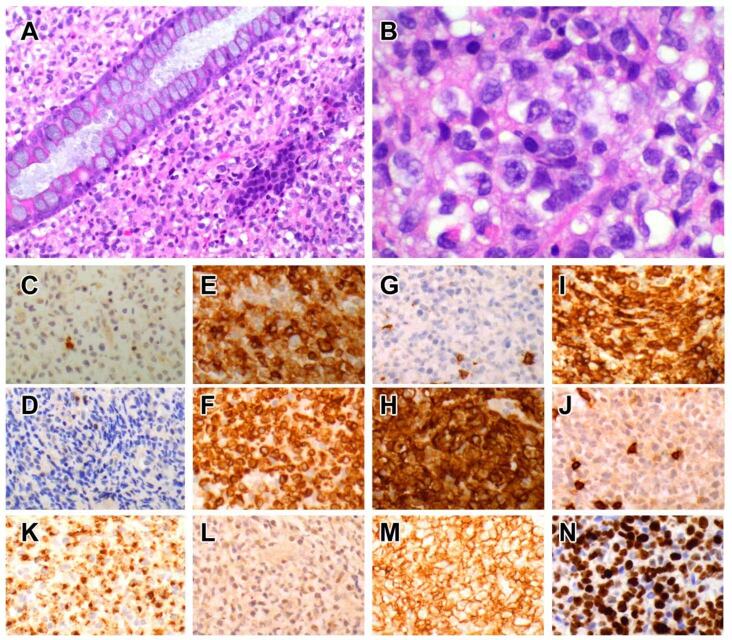
NK-cell gastropathy. The lamina propria is expanded by a dense infiltrate of medium-sized lymphocytes with round nuclei, slightly dispersed chromatin, occasional nucleoli and ample cytoplasm. H&E; original magnification, ×200 (**A**), ×600 (**B**). The lymphoid cell population stains negative for both TCRβ (**C**) and TCRγ (**D**). They stain positive for CD2 (**E**), CD3 (**F**), CD7 (**H**) but not CD5 (**G**). Neoplastic lymphocytes may display CD8αα phenotype, being positive for CD8α (**I**) but not CD8β (**J**). They display a cytotoxic phenotype with TIA1 expression (**K**), but in situ hybridization for EBER is negative (**L**). There is strong expression of CD56 (**M**), and the proliferation fraction is high at 70% with Ki67 staining (**N**).

On endoscopic examination, there are multiple nodules or elevated plaques, with or without erosions or ulcers in the gastrointestinal tract from the oesophagus to the colon [77,78,79,80,81,82,84,85]. The histology is characterized by nodular aggregates of small/medium-sized lymphocytes with bland nuclear features, ample cytoplasm with lightly eosinophilic granular cytoplasm (Figure 1). Diffuse epitheliotropism is not a feature, but focal infiltration of intestinal epithelium has been reported [77,78,79,80,81,82,83,84,85].

#### 5.1.4. Immunophenotype

The CD2+ CD3ε+ CD5− CD7+ CD4− CD8− TCR− CD56+ TIA1+ granzyme B+ immunophenotype [77,78,79,81,82,83,84] is consistent with NK-cells, although Takata has reported rare cases with CD8+ or CD56− phenotypes that displayed similar indolent clinical outcomes [86]. An important point of distinction from extranodal NK/T cell lymphoma and aggressive NK-cell leukaemia is the lack of EBER [77,78,79,80,82,84,86,87]. The proliferation fraction in lymphomatoid gastropathy/NK-cell enteropathy averages around 25% but may be as high as 30–40% [77,82,88].

#### 5.1.5. Cellular Origin from CD56^bright^ Subset of NK-cells

The consistent lack of TCR expression, a polyclonal pattern by T-cell clonality studies and expression of CD56, all point to the origin of this disease from NK-cells within the gastrointestinal mucosa, rather than T-cells that express NK-like features. Takata K et al. reported two cases of lymphomatoid gastropathy that express CD8 by immunohistochemistry [86]. However, this is not inconsistent with the NK-lineage of this entity, as the commonly used antibodies against CD8 in FFPE material are directed against CD8α, and thus, these cases of NK-cell proliferations may well express CD8αα, seen in a subset of NK-cells [41].

All cases of lymphomatoid gastropathy/NK-cell enteropathy are CD56+, and flow cytometry performed in one case showed ‘aberrantly bright’ CD56 expression [84]. Apart from an occasional case that is CD16+ (but with reduction of staining compared to CD56) [79], most cases display dim or negative expression of CD16 [77,85]. The CD2+ CD5− CD7+ phenotype seen in most cases [77,85] is also typical of origin from CD56^bright^ NK-cells (Table 1). Expression of CD335/NKp46 is reported in a case [89], and another case expressed CD94/NKG2 [84], whilst staining for CD103 is negative in a case so tested [76]. This phenotype suggests that lymphomatoid gastropathy/NK-cell enteropathy may arise from the CD56^bright^ subset of NK-cells resident within the lamina propria.

#### 5.1.6. Pathogenetic Mechanisms

The aetiology and pathogenetic mechanisms are unknown. No specific mutations have been described in this entity, but H. pylori infection was often reported in lymphomatoid gastropathy [77,79,80]. A case of NK-cell enteropathy associated with high titres of anti-gliadin antibody showed partial regression following gluten withdrawal, which suggests that the disease may be driven by an abnormal immune response [84]. In addition, clonality has not been demonstrated in this entity (although clonality analysis is difficult to perform in NK-cell neoplasms), and a reactive aetiology has been proposed [84]. However, recurrent mutations of JAK3 have been reported [90], and targeted mutational analysis in a case similarly detected somatic variants of *AXL* and *JAK3* [88], which suggests a neoplastic rather than a reactive process.

### 5.2. Extranodal NK/T Cell Lymphoma, Nasal Type (ENKTL)

#### 5.2.1. Incidence and Prevalence

ENKTL constitutes 3–10% of non-Hodgkin lymphoma in Asia and South America but is uncommon in western populations, where it comprises less than 1% of cases [91]. Gastrointestinal ENKTL is seen in 2.7% of ENKTL and 3.1% of intestinal non-Hodgkin lymphoma cases [92,93]. Primarily seen in Asians and native South Americans, there is an association between certain HLA class II molecules (HLA-DPB1, HLA-DRB1, IL18RAP) and increased risk for this EBV+ tumour [94,95].

#### 5.2.2. Clinical Features

This is an aggressive neoplasm of NK- and cytotoxic T-cells primarily involving the upper aerodigestive tract, skin, testes and gastrointestinal tract.

Gastrointestinal ENKTL primarily affects the small intestine, particularly the ileocaecal region [92,93,96], although another study reported more cases in the colon [97]. It presents at an advanced stage with pain, bleeding, obstruction and bowel perforation [92,96,97,98]. The overall median survival is <8 months [96], despite treatment with curative intent. Adverse prognostic factors include high viral load, tumour perforation, non-nasal site, high stage and poor IPI (international prognostic index) score [92,93,96,99,100].

#### 5.2.3. Pathological Findings

Macroscopically, the disease more often presents as mucosal ulcers or erosions [93,97,98,101] rather than fungating masses [98]. They feature a diffuse population of mainly small/medium-sized cells showing twisted, irregular nuclei, condensed chromatin; with widespread necrosis, karyorrhexis and numerous reactive inflammatory cells [93,101,102]. Occasional cases feature large cells with conspicuous nucleoli [101].

#### 5.2.4. Immunophenotype

In keeping with NK-cell origin in many cases, the tumour stains positive for CD2, CD3ε, CD7, CD56, cytotoxic granules (TIA1, granzyme, perforin), CD158/KIR and CD335/NKp46 but is usually negative for sCD3, CD4, CD8, CD57 and TCR [93,101,102,103,104,105]. Some cases express CD4 [93] or CD8 [93,101], whilst others express TCR of either TCRαβ or TCRγδ types [93], in keeping with T-cell origin. All cases are EBV-associated, but expression of CD103 is uncommon in intestinal ENKTL [106].

#### 5.2.5. Cellular Origin from Activated CD56^bright^ NK-Cells

Many ENKTLs lack TCR expression but display an NK-cell phenotype (CD56+, sCD3−, CD4− CD8−, TIA1+, granzyme B+, perforin+) and lack clonal TCR rearrangement, in keeping with NK-cell lineage.

Similar to CD56^bright^ NK-cells, ENKTLs are CD56+, CD16^dim^, CD57−, CD158/KIR−, CD2+, CD5−, CD335/NKp46+ and CD94+ [105,107,108]. Weak, heterogeneous expression of CD8 due to CD8αα expression is seen in some cases. In addition, ENKTL displays markers of activation, such as HLA-DR, CD45RO, CD7, CD30 and CD3ε [93,100,108,109]. Whilst ENKTL is typically CD4−, rare CD4+ cases have been reported in the gut, skin and nasal sites [93,110,111], but this is in keeping with expression of CD4 in tissue-resident, activated CD56^bright^ NK-cells [42]. In summary, the phenotype of NK-lineage ENKTL indicates an origin from activated CD56^bright^ NK-cells, consistent with EBV-induced transformation of tissue-resident CD56^bright^ NK-cells.

#### 5.2.6. Cellular Origin from CD56+ T-Cells

ENKTL was previously considered a neoplasm of NK-cells. However, it was highlighted by Harabuchi Y [112] that clonal TCR rearrangement can be demonstrated in this tumour. Nagata et al. had created a cell line of nasal ENKTL of TCRγδ lineage [113], and a study in Thailand showed examples of both NK- and T-cell lineages with cases showing expression of TCRαβ, TCRγδ or both. One case was also shown to have clonal rearrangement of *TCRB* gene, in keeping with TCRαβ lineage [114].

Apart from expression of TCR, ENKTL of T-cell lineage was more frequently PDL1+ but less often CD56+ and lacks CXCL13 expression compared to those of NK lineage [114]. CD5 expression has been reported [114,115,116,117] but was seen only in TCRαβ+ cases in one study [114]. In a large study of primary intestinal extranodal NK/T cell lymphoma, the majority (52.9%) was of T-cell lineage with expression of CD4 and CD8 in 14% and 2.4% of cases, respectively. All the CD4+ cases tested showed clonal TCR rearrangements and displayed typical CD56+ EBER+ cytotoxic phenotypes [93].

The cellular origins of ENKTL of T-cell lineage are unclear, but suspicion naturally falls on various subsets of CD56+ T-cells. CD1d-restricted invariant natural killer T-cells (iNKT) produce cytokines within minutes of antigen stimulation, as with NK-cells. Similar to ENKTL, human NKT cells express CD69, CD94/NKG2, CD158/KIR and may be CD4+ CD8+ or CD4− CD8− [118,119]. However, with rare exceptions, NKT cells lack expression of CD335/NKp46 that is typically seen in ENKTL [120]. Distinct from NKT cells is another population of CD56+ mature T-cells in the gut lamina propria that may be of either the CD4+ or CD8+ subset. They express CD45RO, are non-proliferating, and express Th1-like cytokines such as IFNγ and TNFα [121]. Nevertheless, the cellular origin of T-lineage ENKTL remains a matter of debate.

#### 5.2.7. Pathogenetic Mechanisms

Many presumed pathogenetic mechanisms have primarily focused on NK- and T-cell signalling pathways. EBV LMP1 activates NFkB and MAPK, regulates c-myc, induces proliferation and activates survivin. Along with disruption of apoptosis by *TP53* mutations, this may confer a growth advantage [122,123,124]. Dysregulation of *JAK/STAT* (through mutations of *JAK3, STAT3, STAT5B*) [125,126,127], alterations of PDGF, aurora kinase and NF-kB [123,128,129], as well as mutations of *BCOR, MLL2* [130,131], *KIT* [132], *TP53* and *DDX3X* [132,133,134,135], have been reported. Inactivation of tumour suppressor genes (e.g., *PRDM1, PTPRK, FOXO3*) by promoter methylation, truncating mutations or deletion of 6q may also contribute to molecular pathogenesis [128,136,137,138,139]. Immune evasion through *STAT3*-driven upregulation of PDL1 or HLA dysregulation was found to be yet another pathogenetic mechanism [127].

Recently, Xiong et al. described three molecular subtypes of ENKTL with distinct biological characteristics, EBV signatures, clinical outcomes and potential targetable vulnerabilities [140]. The TSIM subtype originates from NK-cells and displays *JAK-STAT* activation and PD-L1 overexpression. Overexpression of c-myc, EBV latency program 1 and poor outcome are characteristics of MB subtype, whilst the HEA subtype originates from T-cells and features histone acetylation and activation of NF-kB. Mutations of *JAK3*, *KCNB2* and *KCNH8* have also been identified in gastrointestinal ENKT, although it is unclear if the mutational landscape is different in other locations.

## 6. Lymphomas and Lymphoproliferative Disorders Derived from T-Cells

Intestinal T-cells give rise to an indolent T-cell lymphoproliferative disorder and three aggressive lymphoma entities. Of the aggressive intestinal T-cell lymphomas, two are characterized by epitheliotropism (probably arising from intraepithelial lymphocytes) and one is non-epitheliotropic (probably arising from a precursor cell in the lamina propria).

### 6.1. Indolent T-Cell Lymphoproliferative Disorder of the GI Tract

#### 6.1.1. Incidence and Prevalence

Based on a recent review, fewer than 80 cases of this uncommon condition have been reported as of early 2021, mostly as case reports and in small series [141].

#### 6.1.2. Clinical Features

This is an indolent, clonal and mostly non-epitheliotropic T-cell lymphoproliferative disorder, which may display CD4+, CD8+, CD4+ CD8+ [142] or CD4− CD8− phenotypes [143,144,145,146]. This is a disease of adults (median age 51.5 in CD4+ and 45 in CD8+ cases) with male predilection [142,143,144,145]. Small intestinal cases typically present with nonspecific symptoms such as dyspepsia, vomiting, and diarrhoea. Some patients complain of ulcers, night sweats, bleeding, malabsorption, weight loss and distension [142,143,144,145,146,147,148,149,150,151,152]. Radiology typically reveals dilated bowel loops with thickened walls. Although enlarged mesenteric nodes may be seen and some cases featuring borderline splenomegaly, the disease is primarily confined to the gut [142,144,145,148,151,153]. Both bowel lesions and enlarged lymph nodes are not avid on PET scans in this disease; thus, this feature can be used to monitor for large cell transformation. A subset of CD4+ cases may progress and disseminate [142,144,154,155], but not CD8+ cases, which are indolent [145,153,156].

#### 6.1.3. Pathological Findings

**Figure 2:**
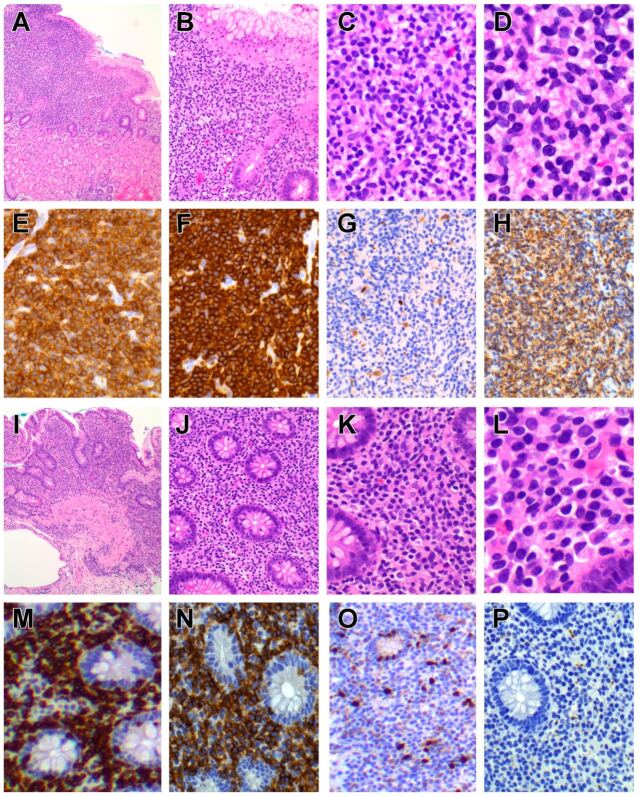
Indolent T-cell lymphoproliferative disorder of CD8+ and CD4+ subtypes. Indolent CD8+ T-cell LPD features a dense lymphocytic infiltrate in the superficial gastric mucosa, comprising sheets of small- to medium-sized lymphocytes with round nuclear contours, condensed chromatin, and scanty cytoplasm. H&E; original magnification, ×40 (**A**), ×100 (**B**), ×200 (**C**), ×400 (**D**). Neoplastic lymphocytes stain positive for CD3 (**E**), CD8 (**F**) with a low proliferation fraction below 10% (**G**) and expression of TIA (**H**). Indolent CD4+ T-cell LPD displays a similar appearance, featuring a dense, non-destructive lymphocytic infiltrate of small- to medium-sized lymphocytes mostly confined to the mucosa. H&E; original magnification, ×40 (**I**), ×100 (**J**), ×200 (**K**), ×400 (**L**). The lymphoid infiltrate stains positive for CD3 (**M**) and CD4 (**N**) with a low proliferation fraction with Ki67 staining (**O**) and lack of CD56 expression (**P**).

The disease presents as thickened mucosa, nodules, polyps or erosions anywhere in the gastrointestinal tract but mainly in the small intestine and colon [145,147,152,156,157]. Histology shows a dense infiltrate of small/medium-sized lymphocytes confined to the lamina propria and submucosa (Figure 2), although focal (but not diffuse) epitheliotropism has been reported, particularly in CD4+ cases, along with varying degrees of villous atrophy [142,144,145,148,152,153,154]. Invasion of the muscularis propria is not a feature [142,144,145], but eosinophils, granulomas and lymphoid follicles may be seen [149,156].

#### 6.1.4. Immunophenotype

Tumours may be of CD4+ or CD8+ subsets. The lymphoid infiltrate is CD2+CD3+ with reduced expression of CD5 and CD7 in CD4+ cases [142,144,147,148,149,152,155], whilst these markers are more likely retained in CD8+ disease [155,156]. Rare CD4−CD8− [152,158] and CD4+ CD8+ cases [152] have been reported, whilst all cases have TCRαβ+ rather than TCRγδ- phenotype [142,144,145,152,155]. The proliferation fraction is low at <10% (particularly in CD8+ cases) [145], and there is no expression of CD335/NKp46 [159].

CD4+ cases lack a cytotoxic phenotype [148,149,155] and stain negative for CD56 [144,148] and CD57 [142]. They lack expression of T-follicular helper (TFH) markers bcl6, CD10 and PD1 [144,148,152], with the exception of rare CD4+ CD8+ and CD4− CD8− cases that may express PD1 [152]. They also stain negative for T-regulatory (Treg) cell markers FoxP3, CD25 [144,152] and the epithelial homing integrin CD103 [142,144,148,152,154]. CD4− CD8− cases may also display aberrant staining for CD20 [158].

CD8+ cases express cytotoxic markers (TIA1+ but usually granzyme B-) [145,155], but apart from few exceptions [152], they usually stain negative for CD56 [160]. A subset of cases also express CD103 [152].

#### 6.1.5. Cellular Origin of Indolent T-Cell LPD of GI Tract

As all cases display clonal rearrangement of TCR genes with TCRαβ+ phenotype, innate lymphoid cell origin can be excluded. The lack of CD56 and CD335/NKp46 expression in most cases does not support an origin from unconventional T-cell subsets with NK-like features. Given that cases may be CD4+ or CD8+ and mostly CD103−, this appears to be a heterogeneous disease arising from conventional T-cells [141,152] in the lamina propria.

##### CD4+ Indolent T-cell LPD

CD4+ cases lack the phenotype of TFH cells (as in primary cutaneous CD4+ small/medium-sized pleomorphic T-cell lymphomas) and Treg cells (as in adult T-cell leukaemia/lymphoma), which lends no support to an origin from these cellular subsets [144]. The disease appears to be a heterogeneous group with cases featuring Th1 (T-bet+), Th2 (GATA3+) and hybrid Th1/2 (T-bet+/GATA3+) differentiation [152]. This suggests that CD4+ indolent T-cell LPD may arise from conventional CD4+ TCRαβ+ T-cells that show variable differentiation towards Th1, Th2 and hybrid Th1/2 types.

##### CD8+ Indolent T-cell LPD

CD8+ cases display a non-activated cytotoxic phenotype and are located in the lamina propria, in keeping with the origin from conventional CD8αβ+ TCRαβ+ T-cells [160].

#### 6.1.6. Pathogenetic Mechanisms

The aetiology is unknown. Given that co-morbidities include H. pylori and viral infections, celiac disease, rheumatic arthritis and Crohn’s disease; antigenic stimulation and immune dysregulation may play a role in pathogenesis. However, TCR genes are clonally rearranged [142,144,145,150,153], and thus, this is clearly a neoplasm. Unlike T-large granular cell leukaemia, *STAT3* mutation is not detected in CD8+ cases [145], but structural alterations in the 3′ UTR region of the IL2 gene have been reported in 50% of cases [152]. CD4+ cases display *STAT3-JAK2* fusions [152,155], mutations of the *JAK-STAT* pathway, mutations of epigenetic modifier genes (*TET2*, *DNMT3A*, *KMT2D)*, *SOCS1* deletion, trisomy 5, t(4;16) translocation involving B-cell maturation antigen (BCMA) and interleukin 2 (IL2) genes, as well as non-recurrent copy number changes [142,144,152,161].

### 6.2. Celiac Disease

Although this review is focused on intestinal NK and T-cell neoplasms, celiac disease is briefly discussed, as it lends understanding towards the cellular origins of EATL, which is a sequela of this disease. Celiac disease is an autoimmune, chronic intestinal disease due to sensitivity to gluten in genetically susceptible individuals, leading to symptoms and signs of malabsorption. Patients will initially respond to a gluten-free diet, but a subset of patients progresses into a refractory celiac disease (RCD) phase that is resistant to gluten withdrawal.

#### 6.2.1. Epidemiology

Celiac disease is more common in western populations, with a prevalence of 0.4% in South America, 0.5% in Africa and North America, 0.6% in Asia, and 0.8% in Europe and Oceania. The global pooled prevalence is 1.4% based on serology and 0.7% based on biopsy results [162].

#### 6.2.2. Clinical Features

The clinical presentation is variable and age-dependent. The typical presentation in children is that of failure to thrive, malabsorption, diarrhoea, abdominal pain and distension, but symptoms may be nonspecific in older patients [163]. Endoscopic features include reduced and scalloped duodenal folds, atypical submucosal vascular pattern (referred to as ‘mosaicism’), as well as mucosal grooves and fissures [164].

#### 6.2.3. Pathological Features

The typical histological features in duodenal biopsies are increased IELs in the proximal third of the villi, reduced goblet cells, thickened basement membrane, crypt hyperplasia, villous blunting and atrophy with varying degrees of severity [165,166]. The increased IELs in celiac disease are CD3+ T-cells of CD4− CD8+ TCRαβ and CD4− CD8− TCRγδ phenotypes [165,166,167,168], but some cells also express CD94/NKG2D [169,170], whilst lymphocytes in the lamina propria are mainly CD4+ CD8− [166,171].

### 6.3. Refractory Celiac Disease (RCD)

When celiac disease does not respond histologically despite 12 months of a gluten-free diet, this is referred to as refractory celiac disease [172]. The absence and presence of phenotypically atypical IELs divides RCD into two phases, RCD1 and RCD2, respectively. About half of those with RCD2 will develop EATL [173,174,175], whilst progression to lymphoma in patients with RCD1 is exceptional [173,176,177]. The histological features of RCD are similar to that of celiac disease, but ulcerative jejunitis and lymphocytic gastritis tend to be more frequent in RCD2 [174].

The IELs in RCD are morphologically normal, but the immunophenotype is atypical in RCD2, being sCD3− but intracytoplasmic CD3+, CD8− and TCRαβ− [178,179,180]. Given that development of EATL is seen almost exclusively in cases with >20% atypical T-cells by flow cytometry [181], the cut-off criterion between RCD1 and RCD2 is defined as >20% of phenotypically atypical (sCD3-, CD3ε+ CD8−) T-cells by flow cytometry [181] and >50% of such T-cells by immunohistochemistry [182,183]. Clonal rearrangement of *TCR* gene is generally negative in RCD1 but is seen in about 75% of RCD2 cases [184,185]. The latter includes clonal rearrangements of *TCRD* and/or *TCRG*, but with either incomplete or no rearrangement of *TCRB* genes, as well as cases that display complete rearrangements of *TCRB*, and *TCRG* and/or *TCRD* [185].

### 6.4. Enteropathy Associated T-Cell Lymphoma

#### 6.4.1. Epidemiology

EATL comprised 4.2% of T-cell lymphomas in one study [186], with an incidence of 0.10/100,000 in the Netherlands [187] and 0.016/100,000 in the United States [188]. In Asia, EATL is rare and comprises only 1.4% (7/490 cases) of T-cell lymphoma cases in a multi-centre Asian study [189]. About half of the cases of EATL are preceded by a history of celiac disease, and the remainder appears de novo [190], although celiac disease may be underdiagnosed.

#### 6.4.2. Clinical Findings

This neoplasm is strongly associated with celiac disease (52% of cases) [190,191,192] and geographical regions with higher frequency of the HLA-DQ2 and DQ8 haplotypes, which are rare in Asia [193]. The disease comprises 5.4% of NK- and T-cell neoplasms [190] and presents in older adults. Usual presentations include abdominal pain, weight loss, diarrhoea or bowel perforation [190,194,195]. Disease spread to lymph nodes, lung, liver and bone marrow occurs in 10–20% of cases, and the median survival is poor at <10 months [157,190,194].

#### 6.4.3. Pathological Findings

**Figure 3:**
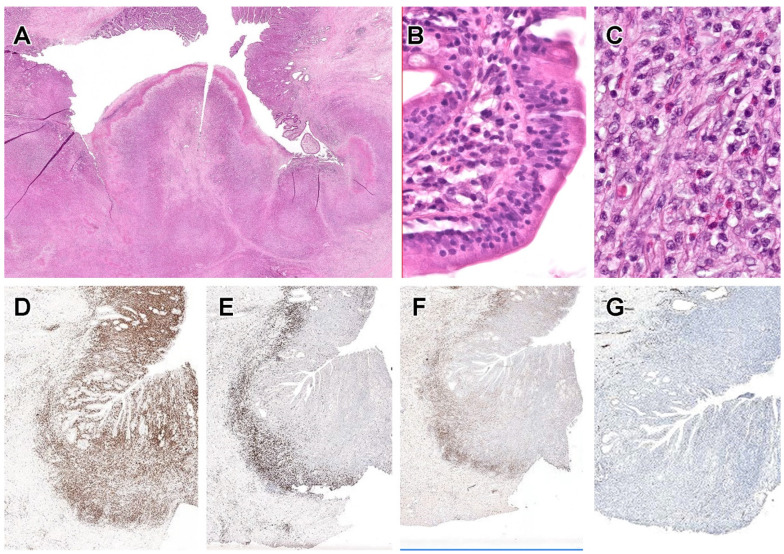
Enteropathy-associated T-cell lymphoma (EATL). An example of EATL showing an invasive, ulcerative tumour with adjacent intestinal mucosa showing fused, stunted villi. H&E; original magnification, ×40 (**A**). Villi showing increased intra-epithelial lymphocytes. H&E; original magnification, ×200 (**B**). Neoplastic lymphocytes display moderate pleomorphism and are accompanied by numerous eosinophils. H&E; original magnification, ×400 (**C**). Intra-epithelial lymphocytes stain positive for CD3 (**D**) but lack expression of CD8 (**E**), CD4 (**F**) and CD56 (**G**).

Typically, there are multiple ulcerated plaques and constricting tumour masses in the small intestine (especially jejunum) and regional lymph nodes [190,191,194]. Microscopic examination features sheets of medium sized to large pleomorphic, immunoblastic or anaplastic lymphocytes showing significant nuclear pleomorphism and necrosis, accompanied by a pronounced inflammatory infiltrate of small lymphocytes, eosinophils, histiocytes and plasma cells (Figure 3). The adjacent intestinal mucosa often shows histological changes of celiac disease such as villous atrophy, crypt hyperplasia and increased IELs [196].

#### 6.4.4. Immunophenotype

Neoplastic lymphocytes are typically CD3ε+ CD4− CD8− TIA1+ granzyme B+ perforin+ CD5− CD7+ CD56− and CD103+, with a high proliferation fraction [180,190,191,194,197,198]. CD56 was detected by flow cytometry in some cases of RCD2 but was absent by immunohistochemistry in the corresponding EATL. Similarly, CD103 expression in EATL may be lost despite CD103+ IELs in the corresponding RCD2 [194]. Expression of TCR is lacking in RCD2 and in most cases of EATL [179,180,191,198], but a case preceded by RCD1 has been reported to arise from TCRγδ+ IELs [199]. There are usually CD30+ large cells within the tumour [194], and there is expression of CD335/NKp46 [159] whilst EBV is negative [194,199]. CD8 is usually negative in EATL, and in one study, it was present only in cases associated with non-clonal enteropathy (celiac disease and RCD1) [194].

#### 6.4.5. Cellular Origins

There are significant differences between RCD1 and RCD2, which may even suggest different disease entities [197] and cellular origins. The prognosis is different with significantly inferior prognosis in RCD2 [177], whilst progression of RCD1 to RCD2 [177] and from RCD1 to EATL [174] is exceptional.

##### Cellular Origin of EATLs Arising from RCD2

Most cases of EATL display a similar phenotype as RCD2, such as CD4− CD8− TCR− CD3ε+ but sCD3−. The phenotype is in keeping with an origin from iCD3+ IELs, which is the group of ILCs that is Id2 independent and rearranges but does not express TCR genes. They are sCD3− and express intracytoplasmic CD3.

##### Cellular Origins of EATL Arising from RCD1 and CD

Whilst EATL with typical CD8− TCR− phenotype usually develops from RCD2, cases associated with RCD1 and celiac disease have been reported that express CD8 and TCR [194,199]. The cellular origin of such exceptional cases is unclear but based on the phenotype, it is reasonable to suggest that they may originate from CD8αβ+ TCRαβ+ (type ‘a’) or CD8αα TCRαβ+/CD8αα+ TCRγδ+ (type ‘b’) IELs.

#### 6.4.6. Pathogenetic Mechanisms in Celiac Disease, RCD and EATL

Gluten molecules penetrate intestinal epithelium by transcytosis or paracellular routes and undergo deamidation by tissue transglutaminase. Deamidated gliadin peptides are presented by dendritic cells in association with HLA DQ2 or DQ8 molecules to CD4+ helper T-cells. Homozygosity for HLA DQ2/8 increases presentation of gliadin peptides [200,201] and portends a higher risk of EATL [202]. Gliadin-specific CD4+ TCRαβ+ IELs mount a Th1 response and produce pro-inflammatory cytokines such as IFNγ, IL2 and IL21 that damage intestinal epithelial cells (IECs) [203].

Gliadin also induces a stress response in intestinal epithelial cells [169,204,205], which strongly express IL15 and MHC class I polypeptide-related sequence A (MICA). Together with IL15-induced NKG2D and DNAX- activation protein 10 (DAP10) expression, these molecules increase intestinal epithelial cell damage, activation and expansion of CD8+ TCRαβ+ IELs [169,205,206,207].

IL15 produced by intestinal epithelial and dendritic cells also enhances the cytotoxicity of IELs through activation of perforin/granzyme B [208]. IL15 upregulates production of IL21 by epithelial cells and CD4+ T-cells [209], further increasing the numbers and cytotoxicity of IELs [210,211].

In early celiac disease, there is an initial increase in CD8αβ+ TCRαβ+ and TCRγδ+ IELs, at the expense of CD7+ TCR- iCD3+ IELs, which normally comprise a minute population of human IELs. However, IL15 suppresses apoptosis of lymphocytes in the intraepithelial compartment, resulting in accumulation of IELs [208,212]. Sustained cytokine stimulation also induces genotoxic stress [213] and acquisition of mutations (*JAK1*, *STAT3*, *STAT5B* and *SOCS1*) that increase sensitivity of IELs to IL15. The result is an expansion and reprogramming towards iCD3+ IELs [25,208,212] that outcompete CD8αβ+ TCRαβ+ IELs [25,212], the result being that iCD3+ IELs become the predominant IEL population in RCD2 and EATL.

The pathogenesis of the rare EATL cases that arise directly from celiac disease or from RCD1 is unclear. Finally, EATLs display clonal rearrangements of *TCRG* and *TCRB* genes. Cytogenetic alterations include gains of 9q31.3-qter, 1q, 5q34-q35.2 and losses of 16q12.1 [214,215]. Frequent mutations reported include members of the JAK-STAT pathway (JAK1, JAK3, STAT3, STAT5B, SOCS1), chromatin modifiers (SETD2, TET2, YLPM1), RAS-pathway (NRAS, KRAS), DNA damage and response (TP53, BCL11B), DAPK1, BBX, TERT, and PRDM1 [216,217].

### 6.5. Monomorphic Epitheliotropic Intestinal T-Cell Lymphoma (MEITL)

#### 6.5.1. Incidence and Prevalence

In the International Peripheral T-cell Lymphoma Project, 5.4% of T-cell lymphomas were either EATL or MEITL with 66% EATL and 34% MEITL cases [190]. In Asia, MEITL appears to be more numerous as a proportion of intestinal T-cell lymphoma, with figures as high as 83% in Japan [218], 80% in Korea [219] and 82% in Hong Kong [220]. A recent Asian study found intestinal T-cell lymphomas to comprise 3.4% of T-cell lymphomas composed of EATL and MEITL in about equal proportions [189]. However, this was not a population-based study and included only cases from selected institutions.

#### 6.5.2. Clinical Features

This is an intestinal T-cell lymphoma that arises from IELs and exhibits epitheliotropism, usually with a CD8+ CD56+ phenotype [221,222]. It primarily arises in older male adults (median age: 58–69) [220,223,224,225,226] and is more commonly reported in Asia, although there is a global distribution [220,222,223,227].

Similar to EATL, patients present with gastrointestinal symptoms such as abdominal pain, diarrhoea, bleeding, weight loss and bowel perforation [220,223,224,225,226,228,229], but the mean duration of symptoms is short (4 months) [224]. The commonest site of involvement is the small intestine [220,222,223,224,225,226], but multifocal lesions are common [220,223,228]. Other than regional lymph nodes, distant dissemination to liver, lung and brain has been reported [222,225,230,231,232,233,234,235]. The prognosis is poor, with a median survival of less than a year [220,223,224,225,226].

#### 6.5.3. Pathological Findings

**Figure 4:**
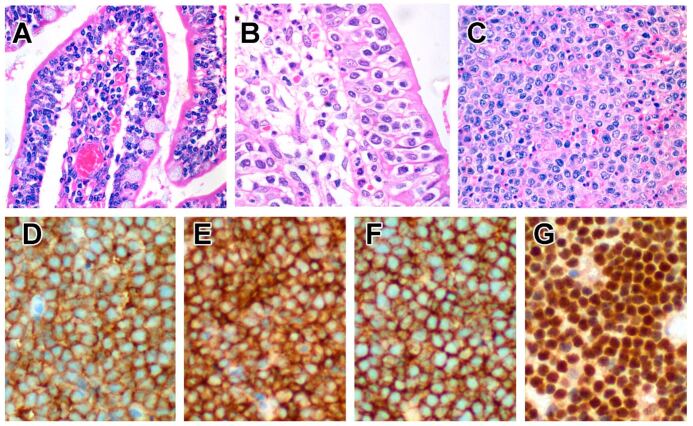
Monomorphic epitheliotropic intestinal T-cell lymphoma (MEITL). A case of MEITL showing small IELs with condensed chromatin in the villi distant from the invasive tumour, H&E; original magnification, ×200 (**A**), whilst atypical lymphocytes in the peripheral zone adjacent to the tumour feature larger nuclei and more open chromatin. H&E; original magnification, ×400 (**B**). Neoplastic lymphocytes in the invasive lymphoma show sheets of monotonous, medium-sized lymphocytes with coarse chromatin. H&E; original magnification, ×200 (**C**). They stain positive for CD3 (**D**), CD8 (**E**), CD56 (**F**), and MATK (**G**).

Endoscopic findings include oedematous and finely granular mucosa, erythematous erosions and shallow ulcers [236,237,238,239,240]. Macroscopically, the tumour presents as a transmural, ulcerative mass, often with perforation. The lymphomatous infiltrate comprises relatively monotonous, medium-sized lymphocytes with scarcity of reactive inflammatory cells and necrosis [191,220,222,225,226,228] (Figure 4). The intestinal mucosa adjacent to the invasive tumour features increased IELs, which may be atypical in morphology and phenotype, whilst IELs appear normal further away from the tumour [220,225], the latter mimicking celiac disease or lymphocytic colitis [228,236,238,239].

#### 6.5.4. Immunophenotype

Neoplastic cells express CD2, CD3 and CD7 but are mostly CD5− [220,221,222,225,226]. Unlike EATL, they express TCRαβ or TCRγδ, with the latter predominating in some series [220,241,242,243]. A number of cases are TCR-silent (although there is rearrangement of TCR genes) [244], and rarely, cases that express both TCRβ and TCRγ have been reported [220,241,242,243,244]. Expression of CD8 and CD56 is typical [245] but not universal, with the majority showing expression of CD8αα homodimers [225]. The proportion of MEITL that expresses CD8αα is likely to be higher, since CD8αα can be induced in CD8αβ+ T-cells [73,74] but cannot be detected by conventional immunohistochemistry. In addition, these tumours are TIA1+ granzyme B+ CD103+ CD335/NKp46+ NKG2D+, and aberrant CD20 expression is common [105,159,214,220,225,226,228,243,245]. Apart from CD8 and TCR expression, another point of distinction from EATL is the expression of MATK [232,246] and Syk [242]. Unlike NKTL, neoplastic cells of MEITL are negative for EBER [220,225,244].

#### 6.5.5. Cellular Origin from Non-Conventional CD8αα+ T-Cells

In addition to CD8 expression, MEITL displays a cytotoxic phenotype (TIA1+, granzyme B+) and expresses NK markers such as CD335/NKp46 and CD56, but unlike EATL, most cases express TCR of either TCRαβ or TCRγδ lineages, in keeping with T-cell origin. There is phenotypic heterogeneity in MEITL, with most cases being CD8αα+TCRαβ+ or CD8αα+TCRγδ+, but there are also cases that are CD8αβ+ TCRαβ+ and a subset that is CD8αα+ but TCR-silent [225]. The central feature is CD8αα expression, which is seen in MEITLs that may be TCRαβ+, TCRγδ+ or TCR-silent [225]. The CD8αα+ TCR+ phenotype in most cases of MEITL is therefore in keeping with the population of type ‘b’ (natural) IELs that are CD8αα+ TCRγδ+ or CD8αα+ TCRαβ+. As for the <25% of MEITLs that are CD8αβ+ TCRαβ+ [225], co-expression of CD8αα cannot be excluded by immunohistochemistry, given that conventional CD8αβ+ TCRαβ+ T-cells can be reprogrammed to express CD8αα. Finally, there is a subset of MEITL that is TCR-silent but displays re-arrangement of TCR genes [244] and are mostly CD8αα+ [225]. This raises the possibility that a minor subset of MEITL originates from iCD3+ IELs that may be either CD8αα- or CD8αα+ (iCD8α IELs).

#### 6.5.6. Pathogenetic Mechanisms

There is rearrangement of TCR genes. Cytogenetic alterations include gains of 9q31.3-qter and losses at 16q12.1, as with EATL [219,243,247]. Alterations in EATL such as gains of 1q32.2-q41 and 5q34-q35.5 were reported by some authors [215,243,244] but not others [214,247]. Other alterations in MEITL include gains of 9q22.31, 4p15.1, 7q34, 8p11.23 and 12p13.31; loss at 7p14.1 [243]; and gains or translocations of *C-MYC* [225,248]. The mutational landscape is quite similar to those seen in EATL, although mutations of *KRAS* and *SETD2* are more common in MEITL [217]. Of higher frequencies are mutations of *SETD2* [217,247,249,250], TP53 [215,217,223,250], KRAS [217,250], members of the *JAK-STAT* [217,247,249,250,251] and G-protein-coupled receptor signalling pathways [247,249]. In keeping with this, miRNA profiling also shows over-representation of JAK-STAT, MAPK and PI3K-AKT pathways [252].

The aetiology is unknown, but there is no association with celiac disease, even in western populations [223]. However, the pathogenesis of MEITL may share overlapping features with that of EATL. Environmental factors such as dietary and microbial stimuli in the intestinal lumen may injure intestinal epithelial cells, leading to production of IL15 as with celiac disease. IL15 triggers T-bet and promotes proliferation, maturation and survival of CD8αα+ natural IELs. However, as with iCD3+ IELs in RCD2 and EATL, CD8αα+ T-cells constitute a small proportion of normal human IELs. Mutations of the *JAK-STAT* pathway and other genetic alterations may facilitate the outgrowth of these rare IEL populations during lymphomagenesis. Similarly, TGFβ, retinoic acid and IFN-γ or IL-27 in the intestinal environment may activate T-bet on CD8αβ+ TCR+ IELs, leading to CD8αα and CD103 expression [8,59,65].

### 6.6. Intestinal T-Cell Lymphoma, NOS

#### 6.6.1. Incidence and Prevalence

As a rare disease and newly recognized entity, accurate incidence and prevalence figures are not available. However, a multi-centre Asian study reported that intestinal T-cell lymphoma, NOS comprises only 0.4% of mature T-cell lymphomas [189].

#### 6.6.2. Clinical Features

By definition, the diagnosis of this intestinal T-cell lymphoma requires the exclusion of other well-defined entities, such as EATL, MEITL, anaplastic large cell lymphoma and ENKTL [192,221]. This neoplasm is more frequently reported in Asia and primarily affects adult males [253]. As this is a diagnosis of exclusion, the aetiology is unknown but is likely to be diverse. Most cases are non-epitheliotropic, but this diagnostic category also includes cases associated with autoimmune enteropathy that shows EATL-like histological features [254].

As with other gastrointestinal tumours, typical presenting complaints include abdominal pain, bleeding and acute abdomen due to bowel perforation. The disease usually involves the small and large intestines but may also arise in the stomach, where hematemesis is a common presentation. Dissemination to regional lymph nodes and distant sites may occur [255,256,257], and the prognosis is poor, but the median survival (35 months) is better than that of EATL and MEITL [92,257]. Large cell morphology is associated with poorer prognosis [256].

#### 6.6.3. Pathological Findings

**Figure 5:**
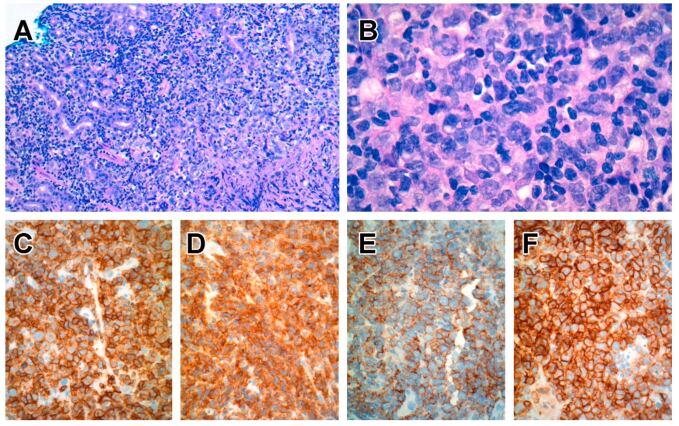
Intestinal T-cell lymphoma (ITCL), NOS involving the stomach. A diffuse lymphomatous infiltrate in the gastric mucosa showing the destruction of foveolar glands. H&E; original magnification, ×40 (**A**). Higher magnification shows medium to large lymphocytes with irregular nuclear contours and occasional prominent nucleoli. H&E; original magnification, ×400 (**B**). Neoplastic lymphocytes stain positive for CD3 (**C**), CD4 (**D**), CD56 (**E**) and CD30 (**F**).

The tumour may be protruding and obstructive or ulcerative and constricting. Neoplastic cells are medium to large lymphocytes that display a greater degree of pleomorphism than that in MEITL (Figure 5). Unlike EATL and MEITL, epitheliotropism is not prominent but may be seen in 15% of cases [256,257]. Neoplastic lymphocytes express CD2 and CD3, with most cases being either CD4+ or CD4− CD8−. A subset displays aberrant expression of CD56, in contrast with indolent CD4+ T-cell LPD of the GI tract. Many cases have a cytotoxic phenotype, being TIA1+, but with variable expression of granzyme B and CD30 [255,256,257]. TCR expression may be either of TCRαβ type or is silent [255,256].

#### 6.6.4. Cellular Origins from Conventional and Non-Conventional T-Cells

As this is a relatively new entity, little data are available to postulate its cellular origins. In addition, as with diagnoses of exclusion, the cellular origins are likely heterogeneous as well.

Most cases are non-epitheliotropic and display expression of TCRαβ+, in keeping with derivation from CD4+TCRαβ+ conventional T-cells in the lamina propria. However, cases that are double negative (CD4−CD8−) and TCR-silent raise the question if they may also arise from innate lymphoid cells in the lamina propria. Cases associated with autoimmune enteropathy that resemble EATL and express TCRαβ may have arisen from conventional type ‘a’ IELs.

#### 6.6.5. Pathogenetic Mechanisms

As with the aetiology, the pathogenesis is unclear. However, mutations of the JAK/STAT and MAPK pathways have been identified [258].

## 7. Treatment and Clinical Trials

### 7.1. Current and Novel Treatment in ENKTL

Unlike most lymphoma types, asparaginase rather than anthracycline-containing regimens are better for the treatment of ENKTL, with or without radiotherapy [259,260,261,262,263]. Hematopoietic stem cell transplantation is indicated if remission is achieved or as salvage therapy [261,264]. In a bid to reduce the toxicity of the currently used SMILE regimen (dexamethasone, methotrexate, ifosfamide, L-asparaginase, etoposide), other asparaginase-containing therapies have been tested [265,266,267,268].

Various novel therapeutic approaches have also been considered [269,270]. These include regimens that target the expression of CD30 and CD38, which are found in some cases of ENKTL [269,271,272,273,274,275]. However, a recent study found no complete responders when treated with daratumumab monotherapy, and the duration of response was short [276]. PDL1 tends to be highly expressed in EBV-associated lymphomas, and immune checkpoint inhibitors have been found to be efficacious in relapsed and refractory ENKTL [277,278,279,280,281]. Clinical trials (which may include other T-cell lymphomas in addition to ENKTL) target phosphatidylinositol 3-kinase (PI3K) with duvelisib (NCT04803201), NFkB with bortezomib [282,283] and the JAK/STAT pathway using inhibitors such as ruxolitinib [284] and tofacitinib (NCT03598959). In addition, several trials using CD7 CAR-T cells (NCT04004637, NCT04033302, NCT04480788) and CD30 CAR-T cells (NCT04008394, NCT03049449) for ENKTL and other T-cell lymphomas are ongoing.

### 7.2. Current and Novel Treatment in EATL, MEITL and ITCL, NOS

The usual treatment for EATL is surgery, followed by anthracycline-containing chemotherapy with or without radiotherapy. In recent years, the Newcastle regimen comprising ifosfamide, etoposide, and epirubicin/methotrexate has shown better outcomes [195,285]. CD30 is often expressed in EATL, and a Phase 2 study found that brentuximab vedotin was efficacious in this tumour [286]. There is an ongoing trial (EATL-001) of the BV-CHP (brentuximab vedotin, cyclophosphamide, doxorubicin, prednisone) regimen followed by consolidation with high dose therapy or transplant (NCT03217643).

Similar to EATL, there is no standard treatment for MEITL, which has a dismal prognosis despite multi-agent chemotherapy. Given its rarity, there are no clinical trial data, although PEG-asparaginase has been reported to be efficacious [287]. ITCL, NOS (as well as EATL and MEITL) are often grouped together with PTCL, NOS in clinical trials, but the best treatment is still unknown. Although cases are usually treated with CHOP (cyclophosphamide, doxorubicin, vincristine, prednisone) as with PTCL, NOS, there is no evidence that anthracyclines make any difference [288]. Combinations of CHOP with etoposide [289,290], alemtuzumab [291,292,293], denileukin diftitox [294] and bortezomib [295,296] have been tried, as well as ACVBP [297], HyperCVAD [298,299] and gemcitabine-containing regimens [300,301,302,303,304]. Novel agents including pralatrexate [305,306], romidepsin [307,308,309], lenalidomide [310,311,312], dasatinib [313,314] and alisertib [315,316,317] have also shown varying degrees of efficacy. Clinical trials of the anti-KIR3DL2 antibody Lacutamab (NCT04984837), immune checkpoint inhibitors (NCT03598998, NCT03240211, NCT03366272), PI3K inhibitor duvelisib (NCT04803201), brentuximab vedotin (NCT02588651, NCT04569032), and pembrolizumab and pralatrexate (NCT03598998) are underway in PTCL, NOS (which may include ENKTL, EATL, MEITL and ITCL, NOS).

## 8. Conclusions

**Figure 6:**
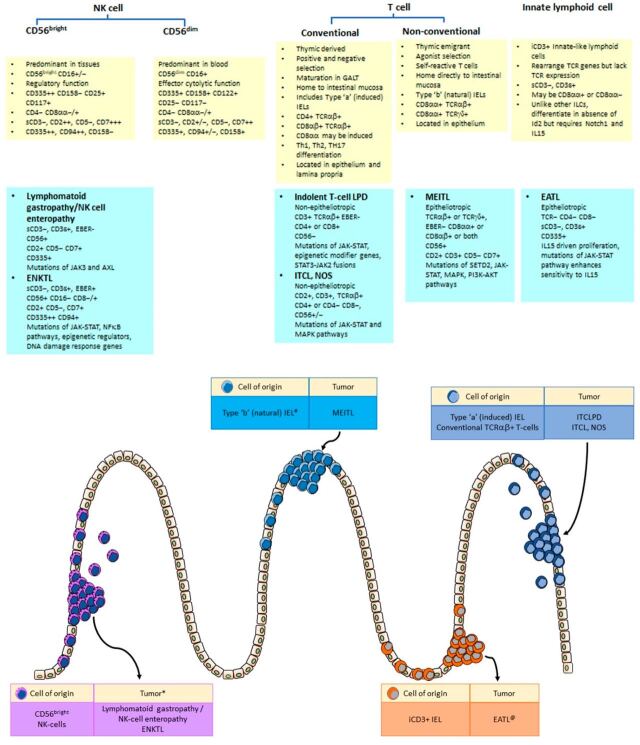
Gastrointestinal NK and T-cell lymphoproliferative disorders with putative cellular origins. Tissue-resident CD56^bright^ NK-cells in intestinal epithelium and lamina propria give rise to both indolent lymphomatoid gastropathy/NK-cell enteropathy and aggressive extranodal NK/T cell αlymphoma. Conventional CD4+ TCRαβ+ and CD8αβ+ TCRαβ+ T-cells are located in the epithelial compartment as IELs and in the lamina propria. They give rise to CD4+ and CD8+ indolent T-cell lymphoproliferative disorders of the GI tract, as well as intestinal T-cell lymphoma, NOS. Unconventional CD8αα+ TCRαβ+ and CD8αα+ TCRγδ+ T-cells constitute a subset of IELs that give rise to MEITL, whilst EATL arises from iCD3+ innate lymphoid cells that express cytoplasmic CD3 but lack sCD3 and TCR. IEL, intraepithelial lymphocyte; MEITL, monomorphic epitheliotrophic intestinal T-cell lymphoma; EATL, enteropathy-associated T-cell lymphoma; ITCLPD, indolent T-cell lymphoproliferative disorder; ITCL NOS, intestinal T-cell lymphoma NOS; ENKTL, extranodal NK/T-cell lymphoma. (* ENKTL of T-cell lineage may arise from CD56+ NKT cells. ^#^ Some cases of MEITL may arise from CD8αα+ CD8αβ+ TCRαβ+ T-cells and iCD8α+ ILCs. ^@^ Cases of EATL that do not progress from RCD2 may arise from conventional CD8αβ+ TCRαβ+ T-cells.).

Lymphoma classification is based on identification of distinct clinico-pathological entities and are often named after the putative cells of origin. The gastrointestinal tract is a unique immunological site where lymphocytes need to serve the dual, conflicting functions of inflammation and tolerance. Complex, specialized subsets of immune cells in the gastrointestinal mucosa have developed unique properties to mount a protective immune reaction against invading microbes yet possess immunomodulatory mechanisms to prevent unwanted tissue damage. Indolent and aggressive lymphoid neoplasms may arise from such immune cells, with unique phenotypes that resemble those of their putative cells of origin (Figure 6).

The two intestinal T-cell lymphomas that demonstrate prominent epitheliotropism, namely EATL and MEITL, are now considered separate entities based on association with celiac disease, morphological features and immunophenotype. Their cellular origins are also different. EATL cases that complicate RCD2 arise from iCD3+ ILCs (and thus not strictly a T-cell lymphoma), whilst MEITL arises from CD8αα+ IELs. However, both iCD3+ ILCs and CD8αα+ IELs (whether CD8αα+ TCRαβ+ T-cells, CD8αα+ TCRγδ+ T-cells or iCD8a+ ILCs) depend on IL15 and T-bet for development and display significant overlap in mutational profiles. At the level of cellular origins and pathogenesis, EATL and MEITL may share more similarities than differences. Future therapeutic strategies may be directed at the microenvironment of the intraepithelial compartment, such as inhibition of IL15 [318]. An understanding of the types and biology of gastrointestinal lymphocytes may hold the key to better diagnosis and treatment of gastrointestinal lymphomas.

## Figures and Tables

**Table 1:** Classification of human innate lymphoid cells [3,9,21].

ILC Subtypes	NK-cell	ILC1	ILC2	ILC3	LTi (Lymphoid Tissue Inducer)
Cytokine production	TH1 cell-associated cytokines: IFNγ		TH2 cell-associated cytokines: IL5, IL13, IL6, IL9	TH17 cell-associated cytokines: IL17, IL22 ^g^	TH17 cell-associated cytokines: IL17, IL22: mainly IL17A+
Phenotype	CD25−/+ ^a^, CD56+, CD117−, CD127−/+, CD161−/+, NKp44−/+ ^b^, NKp46+, ICOS+/− CRTH2−, IL1R−, IL23R−, IL12Rβ2+, ST2−, IL17RB−	CD25+/−, CD56−, CD117−, CD127+ ^d^, CD161+/−, NKp44−, NKp46−, ICOS+, CRTH2−, IL1R+, IL23R−, IL12Rβ2+, ST2−, IL17RB−	CD25+, CD56−, CD117+/−, CD127+, CD161+, NKp44−, NKp46−, ICOS+, CRTH2+, IL1R+, IL23R ND ^f^, IL12Rβ2−, ST2+, IL17RB+	CD25−/+, CD56+/−, CD117+, CD127+, CD161+ ^c^, NKp44+ ^c^, NKp44− ^e^, NKp46+, ICOS+ ^c^, CRTH2 ^c^, IL1R+ ^c^, IL23R+ ^c^, IL12Rβ2− ^c^, ST2− ^c^, IL17RB− ^c^	CD25+/−, CD56−, CD117+, CD127+, CD161+/−, NKp44−, NKp46−, ICOS ND ^f^, CRTH2−, IL1R+, IL23R+, IL12Rβ2−, STR2, IL17RB−
Differentiation of ILCs	T-bet+, Eomes+, RORγt−, GATA3−, AhR−	T-bet+, Eomes−/+, RORγt−, GATA3−, AhR−	T-bet−, Eomes−, RORγt−, RORα+, GATA3+, AhR+	T-bet−, Eomes−, RORγt+, GATA3−, AhR+	T-bet−, Eomes−, RORγt+, GATA3−, AhR+
Function	Pro-inflammatory against bacteria and intracellular viruses		Defence against helmintic infections	Maintain homeostasis, immune tolerance, defence against fungal infections	LTi role in formation of secondary lymphoid organs during embryogenesis

^a^ Expressed in activated CD56^bright^ NK-cells. ^b^ Expressed in activated NK-cells. ^c^ In NCR+ ILC3, ^d^ except for intraepithelial ILC1 cells. ^e^ In NCR- ILC3. ^f^ Not determined. ^g^ NCR+ ILC3 cells primarily express IL22 but less IL17 whilst the reverse is true of NCR- ILC3.

**Table 2:** Phenotype of CD56^bright^ and CD56^dim^ NK-cells.

	CD56^bright^ *	CD56^dim^ *
Functions	Regulatory	Effector cytolytic
Location	Predominantly tissue	Primary in blood
Maturity	Precursor	Mature
NK-cell markers		
CD56	++	+
CD57	−	+
CD16	+/−	++
T cell markers		
CD2	++	+/−
CD5	−	−/+
CD7	+++	++
CD8	−/+ *	−/+ ^@^
Leucocyte integrins		
CD11c	++	+/−
Selectins		
CD62L	++	+/−
Cytokine receptors		
CD122	−/+	+
CD25	+	−
CD4	−/+	−
Growth factor receptor		
CD117	+/−	−
Inhibitory and activating receptors		
KIR/CD158	−	+/−
CD94/NKG2A	++	+/−
ILT2	−	+
Natural cytotoxic receptors		
CD335/NKp46	++	+
Activation markers		
HLA-DR	+/−	−
CD38	+	+
CD26	+	−

* CD56^bright^ and CD56^dim^ refers to strong (bright) and weak (dim) expression of CD56 in immune cells by flow cytometry. ^@^ Whilst most NK-cells lack both CD4 and CD8 expression, CD8 homodimer is expressed in a subset of NK-cells.

## Data Availability

Not applicable.

## References

[1] Kohno S., Ohshima K., Yoneda S., Kodama T., Shirakusa T., Kikuchi M. (2003). Clinicopathological analysis of 143 primary malignant lymphomas in the small and large intestines based on the new WHO classification. Histopathology.

[2] Nakamura S., Matsumoto T., Iida M., Yao T., Tsuneyoshi M. (2003). Primary gastrointestinal lymphoma in Japan: A clinicopathologic analysis of 455 patients with special reference to its time trends. Cancer.

[3] Spits H., Artis D., Colonna M., Diefenbach A., Di Santo J.P., Eberl G., Koyasu S., Locksley R.M., McKenzie A.N., Mebius R.E. (2013). Innate lymphoid cells—A proposal for uniform nomenclature. Nat. Rev. Immunol..

[4] Spits H., Bernink J.H., Lanier L. (2016). NK-cells and type 1 innate lymphoid cells: Partners in host defense. Nat. Immunol..

[5] Spits H., Di Santo J.P. (2011). The expanding family of innate lymphoid cells: Regulators and effectors of immunity and tissue remodeling. Nat. Immunol..

[6] Sun J.C., Lanier L.L. (2011). NK-cell development, homeostasis and function: Parallels with CD8(+) T cells. Nat. Rev. Immunol..

[7] Chen L., Youssef Y., Robinson C., Ernst G.F., Carson M.Y., Young K.A., Scoville S.D., Zhang X., Harris R., Sekhri P. (2018). CD56 Expression Marks Human Group 2 Innate Lymphoid Cell Divergence from a Shared NK-cell and Group 3 Innate Lymphoid Cell Developmental Pathway. Immunity.

[8] Klose C.S., Blatz K., d’Hargues Y., Hernandez P.P., Kofoed-Nielsen M., Ripka J.F., Ebert K., Arnold S.J., Diefenbach A., Palmer E. (2014). The transcription factor T-bet is induced by IL-15 and thymic agonist selection and controls CD8αα(+) intraepithelial lymphocyte development. Immunity.

[9] Vivier E., Artis D., Colonna M., Diefenbach A., Di Santo J.P., Eberl G., Koyasu S., Locksley R.M., McKenzie A.N.J., Mebius R.E. (2018). Innate Lymphoid Cells: 10 Years On. Cell.

[10] Bernink J.H., Krabbendam L., Germar K., de Jong E., Gronke K., Kofoed-Nielsen M., Munneke J.M., Hazenberg M.D., Villaudy J., Buskens C.J. (2015). Interleukin-12 and -23 Control Plasticity of CD127(+) Group 1 and Group 3 Innate Lymphoid Cells in the Intestinal Lamina Propria. Immunity.

[11] Fuchs A., Vermi W., Lee J.S., Lonardi S., Gilfillan S., Newberry R.D., Cella M., Colonna M. (2013). Intraepithelial type 1 innate lymphoid cells are a unique subset of IL-12- and IL-15-responsive IFN-gamma-producing cells. Immunity.

[12] Wong S.H., Walker J.A., Jolin H.E., Drynan L.F., Hams E., Camelo A., Barlow J.L., Neill D.R., Panova V., Koch U. (2012). Transcription factor RORα is critical for nuocyte development. Nat. Immunol..

[13] Mjosberg J.M., Trifari S., Crellin N.K., Peters C.P., van Drunen C.M., Piet B., Fokkens W.J., Cupedo T., Spits H. (2011). Human IL-25- and IL-33-responsive type 2 innate lymphoid cells are defined by expression of CRTH2 and CD161. Nat. Immunol..

[14] Moro K., Kabata H., Tanabe M., Koga S., Takeno N., Mochizuki M., Fukunaga K., Asano K., Betsuyaku T., Koyasu S. (2016). Interferon and IL-27 antagonize the function of group 2 innate lymphoid cells and type 2 innate immune responses. Nat. Immunol..

[15] Neill D.R., Wong S.H., Bellosi A., Flynn R.J., Daly M., Langford T.K., Bucks C., Kane C.M., Fallon P.G., Pannell R. (2010). Nuocytes represent a new innate effector leukocyte that mediates type-2 immunity. Nature.

[16] Cella M., Fuchs A., Vermi W., Facchetti F., Otero K., Lennerz J.K., Doherty J.M., Mills J.C., Colonna M. (2009). A human natural killer cell subset provides an innate source of IL-22 for mucosal immunity. Nature.

[17] Cupedo T., Crellin N.K., Papazian N., Rombouts E.J., Weijer K., Grogan J.L., Fibbe W.E., Cornelissen J.J., Spits H. (2009). Human fetal lymphoid tissue-inducer cells are interleukin 17-producing precursors to RORC+ CD127+ natural killer-like cells. Nat. Immunol..

[18] Hughes T., Becknell B., McClory S., Briercheck E., Freud A.G., Zhang X., Mao H., Nuovo G., Yu J., Caligiuri M.A. (2009). Stage 3 immature human natural killer cells found in secondary lymphoid tissue constitutively and selectively express the TH 17 cytokine interleukin-22. Blood.

[19] van de Pavert S.A. (2021). Lymphoid Tissue inducer (LTi) cell ontogeny and functioning in embryo and adult. Biomed. J..

[20] Wang S., Xia P., Chen Y., Qu Y., Xiong Z., Ye B., Du Y., Tian Y., Yin Z., Xu Z. (2017). Regulatory Innate Lymphoid Cells Control Innate Intestinal Inflammation. Cell.

[21] Hoorweg K., Peters C.P., Cornelissen F., Aparicio-Domingo P., Papazian N., Kazemier G., Mjosberg J.M., Spits H., Cupedo T. (2012). Functional Differences between Human NKp44(-) and NKp44(+) RORC(+) Innate Lymphoid Cells. Front. Immunol..

[22] Simoni Y., Fehlings M., Kløverpris H.N., McGovern N., Koo S.L., Loh C.Y., Lim S., Kurioka A., Fergusson J.R., Tang C.L. (2017). Human Innate Lymphoid Cell Subsets Possess Tissue-Type Based Heterogeneity in Phenotype and Frequency. Immunity.

[23] Talayero P., Mancebo E., Calvo-Pulido J., Rodríguez-Muñoz S., Bernardo I., Laguna-Goya R., Cano-Romero F.L., García-Sesma A., Loinaz C., Jiménez C. (2016). Innate Lymphoid Cells Groups 1 and 3 in the Epithelial Compartment of Functional Human Intestinal Allografts. Am. J. Transplant..

[24] Van Acker A., Gronke K., Biswas A., Martens L., Saeys Y., Filtjens J., Taveirne S., Van Ammel E., Kerre T., Matthys P. (2017). A Murine Intestinal Intraepithelial NKp46-Negative Innate Lymphoid Cell Population Characterized by Group 1 Properties. Cell Rep..

[25] Ettersperger J., Montcuquet N., Malamut G., Guegan N., Lopez-Lastra S., Gayraud S., Reimann C., Vidal E., Cagnard N., Villarese P. (2016). Interleukin-15-Dependent T-Cell-like Innate Intraepithelial Lymphocytes Develop in the Intestine and Transform into Lymphomas in Celiac Disease. Immunity.

[26] Olivares-Villagómez D., Van Kaer L. (2015). iCD8α cells: Living at the edge of the intestinal immune system. Oncotarget.

[27] Van Kaer L., Algood H.M.S., Singh K., Parekh V.V., Greer M.J., Piazuelo M.B., Weitkamp J.H., Matta P., Chaturvedi R., Wilson K.T. (2014). CD8αα(+) innate-type lymphocytes in the intestinal epithelium mediate mucosal immunity. Immunity.

[28] Lanier L.L., Chang C., Spits H., Phillips J.H. (1992). Expression of cytoplasmic CD3 epsilon proteins in activated human adult natural killer (NK) cells and CD3 gamma, delta, epsilon complexes in fetal NK-cells. Implications for the relationship of NK and T lymphocytes. J. Immunol..

[29] Lopez-Verges S., Milush J.M., Pandey S., York V.A., Arakawa-Hoyt J., Pircher H., Norris P.J., Nixon D.F., Lanier L.L. (2010). CD57 defines a functionally distinct population of mature NK-cells in the human CD56dimCD16+ NK-cell subset. Blood.

[30] Beziat V., Duffy D., Quoc S.N., Le Garff-Tavernier M., Decocq J., Combadiere B., Debre P., Vieillard V. (2011). CD56brightCD16+ NK-cells: A functional intermediate stage of NK-cell differentiation. J. Immunol..

[31] Montaldo E., Del Zotto G., Della Chiesa M., Mingari M.C., Moretta A., De Maria A., Moretta L. (2013). Human NK-cell receptors/markers: A tool to analyze NK-cell development, subsets and function. Cytom. Part A.

[32] Romagnani C., Juelke K., Falco M., Morandi B., D’Agostino A., Costa R., Ratto G., Forte G., Carrega P., Lui G. (2007). CD56brightCD16- killer Ig-like receptor- NK-cells display longer telomeres and acquire features of CD56dim NK-cells upon activation. J. Immunol..

[33] Abel A.M., Yang C., Thakar M.S., Malarkannan S. (2018). Natural Killer Cells: Development, Maturation, and Clinical Utilization. Front. Immunol..

[34] Scoville S.D., Freud A.G., Caligiuri M.A. (2017). Modeling Human Natural Killer Cell Development in the Era of Innate Lymphoid Cells. Front. Immunol..

[35] Mattiola I., Pesant M., Tentorio P.F., Molgora M., Marcenaro E., Lugli E., Locati M., Mavilio D. (2015). Priming of Human Resting NK-cells by Autologous M1 Macrophages via the Engagement of IL-1beta, IFN-beta, and IL-15 Pathways. J. Immunol..

[36] Vitale M., Bottino C., Sivori S., Sanseverino L., Castriconi R., Marcenaro E., Augugliaro R., Moretta L., Moretta A. (1998). NKp44, a novel triggering surface molecule specifically expressed by activated natural killer cells, is involved in non-major histocompatibility complex-restricted tumor cell lysis. J. Exp. Med..

[37] Cooper M.A., Fehniger T.A., Turner S.C., Chen K.S., Ghaheri B.A., Ghayur T., Carson W.E., Caligiuri M.A. (2001). Human natural killer cells: A unique innate immunoregulatory role for the CD56(bright) subset. Blood.

[38] Jacobs R., Hintzen G., Kemper A., Beul K., Kempf S., Behrens G., Sykora K.W., Schmidt R.E. (2001). CD56bright cells differ in their KIR repertoire and cytotoxic features from CD56dim NK-cells. Eur. J. Immunol..

[39] Chan A., Hong D.L., Atzberger A., Kollnberger S., Filer A.D., Buckley C.D., McMichael A., Enver T., Bowness P. (2007). CD56bright human NK-cells differentiate into CD56dim cells: Role of contact with peripheral fibroblasts. J. Immunol..

[40] Loza M.J., Perussia B. (2004). The IL-12 signature: NK-cell terminal CD56+high stage and effector functions. J. Immunol..

[41] Geng J., Raghavan M. (2019). CD8αα homodimers function as a coreceptor for KIR3DL1. Proc. Natl. Acad. Sci. USA.

[42] Bernstein H.B., Plasterer M.C., Schiff S.E., Kitchen C.M., Kitchen S., Zack J.A. (2006). CD4 expression on activated NK-cells: Ligation of CD4 induces cytokine expression and cell migration. J. Immunol..

[43] Lugthart G., Melsen J.E., Vervat C., van Ostaijen-Ten Dam M.M., Corver W.E., Roelen D.L., van Bergen J., van Tol M.J., Lankester A.C., Schilham M.W. (2016). Human Lymphoid Tissues Harbor a Distinct CD69+CXCR6+ NK-cell Population. J. Immunol..

[44] Hudspeth K., Donadon M., Cimino M., Pontarini E., Tentorio P., Preti M., Hong M., Bertoletti A., Bicciato S., Invernizzi P. (2016). Human liver-resident CD56(bright)/CD16(neg) NK-cells are retained within hepatic sinusoids via the engagement of CCR5 and CXCR6 pathways. J. Autoimmun..

[45] Melsen J.E., Lugthart G., Lankester A.C., Schilham M.W. (2016). Human Circulating and Tissue-Resident CD56(bright) Natural Killer Cell Populations. Front. Immunol..

[46] Juelke K., Killig M., Luetke-Eversloh M., Parente E., Gruen J., Morandi B., Ferlazzo G., Thiel A., Schmitt-Knosalla I., Romagnani C. (2010). CD62L expression identifies a unique subset of polyfunctional CD56dim NK-cells. Blood.

[47] Poggi A., Benelli R., Vene R., Costa D., Ferrari N., Tosetti F., Zocchi M.R. (2019). Human Gut-Associated Natural Killer Cells in Health and Disease. Front. Immunol..

[48] Parihar R., Dierksheide J., Hu Y., Carson W.E. (2002). IL-12 enhances the natural killer cell cytokine response to Ab-coated tumor cells. J. Clin. Investig..

[49] Artis D., Spits H. (2015). The biology of innate lymphoid cells. Nature.

[50] Ma H., Tao W., Zhu S. (2019). T lymphocytes in the intestinal mucosa: Defense and tolerance. Cell Mol. Immunol..

[51] Leishman A.J., Gapin L., Capone M., Palmer E., MacDonald H.R., Kronenberg M., Cheroutre H. (2002). Precursors of functional MHC class I- or class II-restricted CD8αα(+) T cells are positively selected in the thymus by agonist self-peptides. Immunity.

[52] Qiu Y., Peng K., Liu M., Xiao W., Yang H. (2016). CD8αα TCRalphabeta Intraepithelial Lymphocytes in the Mouse Gut. Dig. Dis. Sci..

[53] Bai L., Peng H. (2018). Generating CD8αα IELs from two sources of thymic precursors. Cell Mol. Immunol..

[54] Ma H., Qiu Y., Yang H. (2021). Intestinal intraepithelial lymphocytes: Maintainers of intestinal immune tolerance and regulators of intestinal immunity. J. Leukoc. Biol..

[55] Cheroutre H., Lambolez F., Mucida D. (2011). The light and dark sides of intestinal intraepithelial lymphocytes. Nat. Rev. Immunol..

[56] Denning T.L., Granger S.W., Mucida D., Graddy R., Leclercq G., Zhang W., Honey K., Rasmussen J.P., Cheroutre H., Rudensky A.Y. (2007). Mouse TCRalphabeta+CD8αα intraepithelial lymphocytes express genes that down-regulate their antigen reactivity and suppress immune responses. J. Immunol..

[57] Olivares-Villagomez D., Van Kaer L. (2018). Intestinal Intraepithelial Lymphocytes: Sentinels of the Mucosal Barrier. Trends Immunol..

[58] Van Kaer L., Olivares-Villagomez D. (2018). Development, Homeostasis, and Functions of Intestinal Intraepithelial Lymphocytes. J. Immunol..

[59] Reis B.S., Hoytema van Konijnenburg D.P., Grivennikov S.I., Mucida D. (2014). Transcription factor T-bet regulates intraepithelial lymphocyte functional maturation. Immunity.

[60] Das G., Augustine M.M., Das J., Bottomly K., Ray P., Ray A. (2003). An important regulatory role for CD4+CD8 alpha alpha T cells in the intestinal epithelial layer in the prevention of inflammatory bowel disease. Proc. Natl. Acad. Sci. USA.

[61] Zhou C., Qiu Y., Yang H. (2019). CD4CD8αα IELs: They Have Something to Say. Front. Immunol..

[62] He X., He X., Dave V.P., Zhang Y., Hua X., Nicolas E., Xu W., Roe B.A., Kappes D.J. (2005). The zinc finger transcription factor Th-POK regulates CD4 versus CD8 T-cell lineage commitment. Nature.

[63] Taniuchi I., Osato M., Egawa T., Sunshine M.J., Bae S.C., Komori T., Ito Y., Littman D.R. (2002). Differential requirements for Runx proteins in CD4 repression and epigenetic silencing during T lymphocyte development. Cell.

[64] Cervantes-Barragan L., Chai J.N., Tianero M.D., Di Luccia B., Ahern P.P., Merriman J., Cortez V.S., Caparon M.G., Donia M.S., Gilfillan S. (2017). Lactobacillus reuteri induces gut intraepithelial CD4(+)CD8αα(+) T cells. Science.

[65] Kwong B., Lazarevic V. (2014). T-bet orchestrates CD8αα IEL differentiation. Immunity.

[66] Masopust D., Vezys V., Wherry E.J., Barber D.L., Ahmed R. (2006). Cutting edge: Gut microenvironment promotes differentiation of a unique memory CD8 T cell population. J. Immunol..

[67] Konkel J.E., Maruyama T., Carpenter A.C., Xiong Y., Zamarron B.F., Hall B.E., Kulkarni A.B., Zhang P., Bosselut R., Chen W. (2011). Control of the development of CD8αα+ intestinal intraepithelial lymphocytes by TGF-beta. Nat. Immunol..

[68] Cervantes-Barragan L., Colonna M. (2018). AHR signaling in the development and function of intestinal immune cells and beyond. Semin. ImmunoPathol..

[69] Yu S., Bruce D., Froicu M., Weaver V., Cantorna M.T. (2008). Failure of T cell homing, reduced CD4/CD8αα intraepithelial lymphocytes, and inflammation in the gut of vitamin D receptor KO mice. Proc. Natl. Acad. Sci. USA.

[70] Mayassi T., Jabri B. (2018). Human intraepithelial lymphocytes. Mucosal Immunol..

[71] Bhagat G., Naiyer A.J., Shah J.G., Harper J., Jabri B., Wang T.C., Green P.H., Manavalan J.S. (2008). Small intestinal CD8+TCRgammadelta+NKG2A+ intraepithelial lymphocytes have attributes of regulatory cells in patients with celiac disease. J. Clin. Investig..

[72] Mikulak J., Oriolo F., Bruni E., Roberto A., Colombo F.S., Villa A., Bosticardo M., Bortolomai I., Lo Presti E., Meraviglia S. (2019). NKp46-expressing human gut-resident intraepithelial Vdelta1 T cell subpopulation exhibits high antitumor activity against colorectal cancer. JCI Insight.

[73] Cawthon A.G., Alexander-Miller M.A. (2002). Optimal colocalization of TCR and CD8 as a novel mechanism for the control of functional avidity. J. Immunol..

[74] Cawthon A.G., Lu H., Alexander-Miller M.A. (2001). Peptide requirement for CTL activation reflects the sensitivity to CD3 engagement: Correlation with CD8alphabeta versus CD8αα expression. J. Immunol..

[75] Cheroutre H., Lambolez F. (2008). Doubting the TCR coreceptor function of CD8αα. Immunity.

[76] Xia D., Morgan E.A., Berger D., Pinkus G.S., Ferry J.A., Zukerberg L.R. (2019). NK-Cell Enteropathy and Similar Indolent Lymphoproliferative Disorders: A Case Series With Literature Review. Am. J. Clin. Pathol..

[77] Takeuchi K., Yokoyama M., Ishizawa S., Terui Y., Nomura K., Marutsuka K., Nunomura M., Fukushima N., Yagyuu T., Nakamine H. (2010). Lymphomatoid gastropathy: A distinct clinicopathologic entity of self-limited pseudomalignant NK-cell proliferation. Blood.

[78] Ishibashi Y., Matsuzono E., Yokoyama F., Ohara Y., Sugai N., Seki H., Miura A., Fujita J., Suzuki J., Fujisawa T. (2013). A case of lymphomatoid gastropathy: A self-limited pseudomalignant natural killer (NK)-cell proliferative disease mimicking NK/T-cell lymphomas. Clin. J. Gastroenterol..

[79] Tanaka T., Megahed N., Takata K., Asano N., Niwa Y., Hirooka Y., Goto H. (2011). A case of lymphomatoid gastropathy: An indolent CD56-positive atypical gastric lymphoid proliferation, mimicking aggressive NK/T cell lymphomas. Pathol. Res. Pract..

[80] Terai T., Sugimoto M., Uozaki H., Kitagawa T., Kinoshita M., Baba S., Yamada T., Osawa S., Sugimoto K. (2012). Lymphomatoidgastropathy mimicking extranodal NK/T cell lymphoma, nasal type: A case report. World J. Gastroenterol..

[81] Koh J., Go H., Lee W.A., Jeon Y.K. (2014). Benign Indolent CD56-Positive NK-Cell Lymphoproliferative Lesion Involving Gastrointestinal Tract in an Adolescent. Korean J. Pathol..

[82] Mansoor A., Pittaluga S., Beck P.L., Wilson W.H., Ferry J.A., Jaffe E.S. (2011). NK-cell enteropathy: A benign NK-cell lymphoproliferative disease mimicking intestinal lymphoma: Clinicopathologic features and follow-up in a unique case series. Blood.

[83] McElroy M.K., Read W.L., Harmon G.S., Weidner N. (2011). A unique case of an indolent CD56-positive T-cell lymphoproliferative disorder of the gastrointestinal tract: A lesion potentially misdiagnosed as natural killer/T-cell lymphoma. Ann. Diagn. Pathol..

[84] Vega F., Chang C.C., Schwartz M.R., Preti H.A., Younes M., Ewton A., Verm R., Jaffe E.S. (2006). Atypical NK-cell proliferation of the gastrointestinal tract in a patient with antigliadin antibodies but not celiac disease. Am. J. Surg. Pathol..

[85] Isom J.A., Arroyo M.R., Reddy D., Joshi-Guske P., Al-Quran S.Z., Li Y., Allan R.W. (2018). NK-cell enteropathy: A case report with 10 years of indolent clinical behaviour. Histopathology.

[86] Takata K., Noujima-Harada M., Miyata-Takata T., Ichimura K., Sato Y., Miyata T., Naruse K., Iwamoto T., Tari A., Masunari T. (2015). Clinicopathologic analysis of 6 lymphomatoid gastropathy cases: Expanding the disease spectrum to CD4-CD8+ cases. Am. J. Surg. Pathol..

[87] Yamamoto J., Fujishima F., Ichinohasama R., Imatani A., Asano N., Harigae H. (2011). A case of benign natural killer cell proliferative disorder of the stomach (gastric manifestation of natural killer cell lymphomatoid gastroenteropathy) mimicking extranodal natural killer/T-cell lymphoma. Leuk. Lymphoma.

[88] Montes-Moreno S., King R.L., Oschlies I., Ponzoni M., Goodlad J.R., Dotlic S., Traverse-Glehen A., Ott G., Ferry J.A., Calaminici M. (2020). Update on lymphoproliferative disorders of the gastrointestinal tract: Disease spectrum from indolent lymphoproliferations to aggressive lymphomas. Virchows Arch..

[89] Freud A.G., Zhao S., Wei S., Gitana G.M., Molina-Kirsch H.F., Atwater S.K., Natkunam Y. (2013). Expression of the activating receptor, NKp46 (CD335), in human natural killer and T-cell neoplasia. Am. J. Clin. Pathol..

[90] Xiao W., Gupta G.K., Yao J., Jang Y.J., Xi L., Baik J., Sigler A., Kumar A., Moskowitz A.J., Arcila M.E. (2019). Recurrent somatic JAK3 mutations in NK-cell enteropathy. Blood.

[91] Harabuchi Y., Takahara M., Kishibe K., Nagato T., Kumai T. (2019). Extranodal Natural Killer/T-Cell Lymphoma, Nasal Type: Basic Science and Clinical Progress. Front. Pediatr..

[92] Kim S.J., Choi C.W., Mun Y.C., Oh S.Y., Kang H.J., Lee S.I., Won J.H., Kim M.K., Kwon J.H., Kim J.S. (2011). Multicenter retrospective analysis of 581 patients with primary intestinal non-hodgkin lymphoma from the Consortium for Improving Survival of Lymphoma (CISL). BMC Cancer.

[93] Yu B.H., Shui R.H., Sheng W.Q., Wang C.F., Lu H.F., Zhou X.Y., Zhu X.Z., Li X.Q. (2016). Primary Intestinal Extranodal Natural Killer/T-Cell Lymphoma, Nasal.l Type: A Comprehensive Clinicopathological Analysis of 55 Cases. PLoS ONE.

[94] Li Z., Xia Y., Feng L.N., Chen J.R., Li H.M., Cui J., Cai Q.Q., Sim K.S., Nairismagi M.L., Laurensia Y. (2016). Genetic risk of extranodal natural killer T-cell lymphoma: A genome-wide association study. Lancet Oncol..

[95] Lin G.W., Xu C., Chen K., Huang H.Q., Chen J., Song B., Chan J.K.C., Li W., Liu W., Shih L.Y. (2020). Genetic risk of extranodal natural killer T-cell lymphoma: A genome-wide association study in multiple populations. Lancet Oncol..

[96] Kim S.J., Jung H.A., Chuang S.S., Hong H., Guo C.C., Cao J., Hong X.N., Suzuki R., Kang H.J., Won J.H. (2013). Extranodal natural killer/T-cell lymphoma involving the gastrointestinal tract: Analysis of clinical features and outcomes from the Asia Lymphoma Study Group. J. Hematol. Oncol..

[97] Jiang M., Chen X., Yi Z., Zhang X., Zhang B., Luo F., Jiang Y., Zou L. (2013). Prognostic characteristics of gastrointestinal tract NK/T-cell lymphoma: An analysis of 47 patients in China. J. Clin. Gastroenterol..

[98] Kim J.H., Lee J.H., Lee J., Oh S.O., Chang D.K., Rhee P.L., Kim J.J., Rhee J.C., Lee J., Kim W.S. (2007). Primary NK-/T-cell lymphoma of the gastrointestinal tract: Clinical characteristics and endoscopic findings. Endoscopy.

[99] Lee G., Ryu H.J., Choi J.W., Kang H., Yang W.I., Yang I.S., Seo M.K., Kim S., Yoon S.O. (2019). Characteristic gene alterations in primary gastrointestinal T- and NK-cell lymphomas. Leukemia.

[100] Suzuki R., Suzumiya J., Yamaguchi M., Nakamura S., Kameoka J., Kojima H., Abe M., Kinoshita T., Yoshino T., Iwatsuki K. (2010). Prognostic factors for mature natural killer (NK) cell neoplasms: Aggressive NK-cell leukemia and extranodal NK-cell lymphoma, nasal type. Ann. Oncol..

[101] Tang X.F., Yang L., Duan S., Guo H., Guo Q.N. (2018). Intestinal T-cell and NK/T-cell lymphomas: A clinicopathological study of 27 Chinese patients. Ann. Diagn. Pathol..

[102] Chan J.K.C., Quintanilla-Martinez L., Ferry J.A., Swerdlow S.H., Campo E., Harris N.L., Jaffe E.S., Pileri S.A., Stein H., Thiele J., Arber D.A., Hasserjian R.P., Le Beau M.M. (2017). Extranodal NK/T-cell lymphoma, nasal type. WHO Classification of Tumours of Haematopoietic and Lymphoid Tissues.

[103] Ko Y.H., Li G.D., Takeuchi K., WHO Classification of Tumours Editorial Board (2019). Extranodal NK/T cell lymphoma. Digestive System Tumours.

[104] Dukers D.F., Vermeer M.H., Jaspars L.H., Sander C.A., Flaig M.J., Vos W., Willemze R., Meijer C.J. (2001). Expression of killer cell inhibitory receptors is restricted to true NK-cell lymphomas and a subset of intestinal enteropathy-type T cell lymphomas with a cytotoxic phenotype. J. Clin. Pathol..

[105] Uemura Y., Isobe Y., Uchida A., Asano J., Nishio Y., Sakai H., Hoshikawa M., Takagi M., Nakamura N., Miura I. (2018). Expression of activating natural killer-cell receptors is a hallmark of the innate-like T-cell neoplasm in peripheral T-cell lymphomas. Cancer Sci..

[106] Ishibashi H., Nimura S., Ishitsuka K., Mihashi Y., Mizoguchi M., Nakamura S., Okamura S., Momosaki S., Aoyagi K., Sakisaka S. (2016). High Expression of Intestinal Homing Receptor CD103 in Adult T-Cell Leukemia/Lymphoma, Similar to 2 Other CD8+ T-Cell Lymphomas. Am. J. Surg. Pathol..

[107] de Mel S., Li J.B., Abid M.B., Tang T. (2018). The utility of flow cytometry in differentiating NK/T cell lymphoma from indolent and reactive NK-cell proliferations. Cytom. Part. B Clin. Cytom..

[108] Lima M. (2015). Extranodal NK/T cell lymphoma and aggressive NK-cell leukaemia: Evidence for their origin on CD56+bright CD16-/+dim NK-cells. Pathology.

[109] Au W.Y., Weisenburger D.D., Intragumtornchai T., Nakamura S., Kim W.S., Sng I., Vose J., Armitage J.O., Liang R., International Peripheral T.C.L.P. (2009). Clinical differences between nasal and extranasal natural killer/T-cell lymphoma: A study of 136 cases from the International Peripheral T-Cell Lymphoma Project. Blood.

[110] Liu S., Zhou X., Song A., Huo Z., Wang Y., Liu Y. (2019). Nasal-type extranodal natural killer/T-cell lymphoma presenting with a mass on the buttock: A case report. Medicine.

[111] Ng S.B., Lai K.W., Murugaya S., Lee K.M., Loong S.L., Fook-Chong S., Tao M., Sng I. (2004). Nasal-type extranodal natural killer/T-cell lymphomas: A clinicopathologic and genotypic study of 42 cases in Singapore. Mod. Pathol..

[112] Harabuchi Y., Imai S., Wakashima J., Hirao M., Kataura A., Osato T., Kon S. (1996). Nasal T-cell lymphoma causally associated with Epstein-Barr virus: Clinicopathologic, phenotypic, and genotypic studies. Cancer.

[113] Nagata H., Konno A., Kimura N., Zhang Y., Kimura M., Demachi A., Sekine T., Yamamoto K., Shimizu N. (2001). Characterization of novel natural killer (NK)-cell and gammadelta T-cell lines established from primary lesions of nasal T/NK-cell lymphomas associated with the Epstein-Barr virus. Blood.

[114] Pongpruttipan T., Sukpanichnant S., Assanasen T., Wannakrairot P., Boonsakan P., Kanoksil W., Kayasut K., Mitarnun W., Khuhapinant A., Bunworasate U. (2012). Extranodal NK/T-cell lymphoma, nasal type, includes cases of natural killer cell and alphabeta, gammadelta, and alphabeta/gammadelta T-cell origin: A comprehensive clinicopathologic and phenotypic study. Am. J. Surg. Pathol..

[115] Au W.Y., Ma S.Y., Chim C.S., Choy C., Loong F., Lie A.K., Lam C.C., Leung A.Y., Tse E., Yau C.C. (2005). Clinicopathologic features and treatment outcome of mature T-cell and natural killer-cell lymphomas diagnosed according to the World Health Organization classification scheme: A single center experience of 10 years. Ann. Oncol..

[116] Gaal K., Sun N.C., Hernandez A.M., Arber D.A. (2000). Sinonasal NK/T-cell lymphomas in the United States. Am. J. Surg. Pathol..

[117] Schwartz E.J., Molina-Kirsch H., Zhao S., Marinelli R.J., Warnke R.A., Natkunam Y. (2008). Immunohistochemical characterization of nasal-type extranodal NK/T-cell lymphoma using a tissue microarray: An analysis of 84 cases. Am. J. Clin. Pathol..

[118] Heller N.B.-B.R., Naler L., Sen J.M., Soboloff J.K.D. (2018). Natural Killer T (NKT) Cells in Mice and Men. Signaling Mechanisms Regulating T Cell Diversity and Function.

[119] Krovi S.H., Gapin L. (2018). Invariant Natural Killer T Cell Subsets—More Than Just Developmental Intermediates. Front. Immunol..

[120] Yu J., Mitsui T., Wei M., Mao H., Butchar J.P., Shah M.V., Zhang J., Mishra A., Alvarez-Breckenridge C., Liu X. (2011). NKp46 identifies an NKT cell subset susceptible to leukemic transformation in mouse and human. J. Clin. Investig..

[121] Cohavy O., Targan S.R. (2007). CD56.6 Marks an Effector T Cell Subset in the Human Intestine. J. Immunol..

[122] de Mel S., Hue S.S., Jeyasekharan A.D., Chng W.J., Ng S.B. (2019). Molecular pathogenic pathways in extranodal NK/T cell lymphoma. J. Hematol. Oncol..

[123] Ng S.B., Selvarajan V., Huang G., Zhou J., Feldman A.L., Law M., Kwong Y.L., Shimizu N., Kagami Y., Aozasa K. (2011). Activated oncogenic pathways and therapeutic targets in extranodal nasal-type NK/T cell lymphoma revealed by gene expression profiling. J. Pathol..

[124] Saleem A., Natkunam Y. (2020). Extranodal NK/T-Cell Lymphomas: The Role of Natural Killer Cells and EBV in Lymphomagenesis. Int. J. Mol. Sci..

[125] Bouchekioua A., Scourzic L., de Wever O., Zhang Y., Cervera P., Aline-Fardin A., Mercher T., Gaulard P., Nyga R., Jeziorowska D. (2014). JAK3 deregulation by activating mutations confers invasive growth advantage in extranodal nasal-type natural killer cell lymphoma. Leukemia.

[126] Koo G.C., Tan S.Y., Tang T., Poon S.L., Allen G.E., Tan L., Chong S.C., Ong W.S., Tay K., Tao M. (2012). Janus kinase 3-activating mutations identified in natural killer/T-cell lymphoma. Cancer Discov..

[127] Song T.L., Nairismagi M.L., Laurensia Y., Lim J.Q., Tan J., Li Z.M., Pang W.L., Kizhakeyil A., Wijaya G.C., Huang D.C. (2018). Oncogenic activation of the STAT3 pathway drives PD-L1 expression in natural killer/T-cell lymphoma. Blood.

[128] Huang Y., de Reynies A., de Leval L., Ghazi B., Martin-Garcia N., Travert M., Bosq J., Briere J., Petit B., Thomas E. (2010). Gene expression profiling identifies emerging oncogenic pathways operating in extranodal NK/T-cell lymphoma, nasal type. Blood.

[129] Iqbal J., Weisenburger D.D., Chowdhury A., Tsai M.Y., Srivastava G., Greiner T.C., Kucuk C., Deffenbacher K., Vose J., Smith L. (2011). Natural killer cell lymphoma shares strikingly similar molecular features with a group of non-hepatosplenic gammadelta T-cell lymphoma and is highly sensitive to a novel aurora kinase A inhibitor in vitro. Leukemia.

[130] Dobashi A., Tsuyama N., Asaka R., Togashi Y., Ueda K., Sakata S., Baba S., Sakamoto K., Hatake K., Takeuchi K. (2016). Frequent BCOR aberrations in extranodal NK/T-Cell lymphoma, nasal type. Genes Chromosomes Cancer.

[131] Lee S., Park H.Y., Kang S.Y., Kim S.J., Hwang J., Lee S., Kwak S.H., Park K.S., Yoo H.Y., Kim W.S. (2015). Genetic alterations of JAK/STAT cascade and histone modification in extranodal NK/T-cell lymphoma nasal type. Oncotarget.

[132] Hongyo T., Li T., Syaifudin M., Baskar R., Ikeda H., Kanakura Y., Aozasa K., Nomura T. (2000). Specific c-kit mutations in sinonasal natural killer/T-cell lymphoma in China and Japan. Cancer Res..

[133] de Mel S., Soon G.S., Mok Y., Chung T.H., Jeyasekharan A.D., Chng W.J., Ng S.B. (2018). The Genomics and Molecular Biology of Natural Killer/T-Cell Lymphoma: Opportunities for Translation. Int. J. Mol. Sci..

[134] Jiang L., Gu Z.H., Yan Z.X., Zhao X., Xie Y.Y., Zhang Z.G., Pan C.M., Hu Y., Cai C.P., Dong Y. (2015). Exome sequencing identifies somatic mutations of DDX3X in natural killer/T-cell lymphoma. Nat. Genet..

[135] Quintanilla-Martinez L., Kremer M., Keller G., Nathrath M., Gamboa-Dominguez A., Meneses A., Luna-Contreras L., Cabras A., Hoefler H., Mohar A. (2001). p53 Mutations in nasal natural killer/T-cell lymphoma from Mexico: Association with large cell morphology and advanced disease. Am. J. Pathol..

[136] Chen Y.W., Guo T., Shen L., Wong K.Y., Tao Q., Choi W.W., Au-Yeung R.K., Chan Y.P., Wong M.L., Tang J.C. (2015). Receptor-type tyrosine-protein phosphatase kappa directly targets STAT3 activation for tumor suppression in nasal NK/T-cell lymphoma. Blood.

[137] Iqbal J., Kucuk C., Deleeuw R.J., Srivastava G., Tam W., Geng H., Klinkebiel D., Christman J.K., Patel K., Cao K. (2009). Genomic analyses reveal global functional alterations that promote tumor growth and novel tumor suppressor genes in natural killer-cell malignancies. Leukemia.

[138] Karube K., Nakagawa M., Tsuzuki S., Takeuchi I., Honma K., Nakashima Y., Shimizu N., Ko Y.H., Morishima Y., Ohshima K. (2011). Identification of FOXO3 and PRDM1 as tumor-suppressor gene candidates in NK-cell neoplasms by genomic and functional analyses. Blood.

[139] Zhang Z., Liang L., Li D., Nong L., Liu J., Qu L., Zheng Y., Zhang B., Li T. (2017). Hypermethylation of PRDM1/Blimp-1 promoter in extranodal NK/T-cell lymphoma, nasal type: An evidence of predominant role in its downregulation. Hematol. Oncol..

[140] Xiong J., Cui B.W., Wang N., Dai Y.T., Zhang H., Wang C.F., Zhong H.J., Cheng S., Ou-Yang B.S., Hu Y. (2020). Genomic and Transcriptomic Characterization of Natural Killer T Cell Lymphoma. Cancer Cell.

[141] Sanguedolce F., Zanelli M., Zizzo M., Luminari S., Martino G., Soriano A., Ricci L., Caprera C., Ascani S. (2021). Indolent T-Cell Lymphoproliferative Disorders of the Gastrointestinal Tract (iTLPD-GI): A Review. Cancers.

[142] Carbonnel F., d’Almagne H., Lavergne A., Matuchansky C., Brouet J.C., Sigaux F., Beaugerie L., Nemeth J., Coffin B., Cosnes J. (1999). The clinicopathological features of extensive small intestinal CD4 T cell infiltration. Gut.

[143] Egawa N., Fukayama M., Kawaguchi K., Hishima T., Hayashi Y., Funata N., Ibuka T., Koike M., Miyashita H., Tajima T. (1995). Relapsing oral and colonic ulcers with monoclonal T-cell infiltration. A low grade mucosal T-lymphoproliferative disease of the digestive tract. Cancer.

[144] Margolskee E., Jobanputra V., Lewis S.K., Alobeid B., Green P.H., Bhagat G. (2013). Indolent small intestinal CD4+ T-cell lymphoma is a distinct entity with unique biologic and clinical features. PLoS ONE.

[145] Perry A.M., Warnke R.A., Hu Q., Gaulard P., Copie-Bergman C., Alkan S., Wang H.Y., Cheng J.X., Bacon C.M., Delabie J. (2013). Indolent T-cell lymphoproliferative disease of the gastrointestinal tract. Blood.

[146] Carbonnel F., Lavergne A., Messing B., Tsapis A., Berger R., Galian A., Nemeth J., Brouet J.C., Rambaud J.C. (1994). Extensive small intestinal T-cell lymphoma of low-grade malignancy associated with a new chromosomal translocation. Cancer.

[147] Hirakawa K., Fuchigami T., Nakamura S., Daimaru Y., Ohshima K., Sakai Y., Ichimaru T. (1996). Primary gastrointestinal T-cell lymphoma resembling multiple lymphomatous polyposis. Gastroenterology.

[148] Sena Teixeira Mendes L., Attygalle A.D., Cunningham D., Benson M., Andreyev J., Gonzales-de-Castro D., Wotherspoon A. (2014). CD4-positive small T-cell lymphoma of the intestine presenting with severe bile-acid malabsorption: A supportive symptom control approach. Br. J. Haematol..

[149] Svrcek M., Garderet L., Sebbagh V., Rosenzwajg M., Parc Y., Lagrange M., Bennis M., Lavergne-Slove A., Flejou J.F., Fabiani B. (2007). Small intestinal CD4+ T-cell lymphoma: A rare distinctive clinicopathological entity associated with prolonged survival. Virchows Arch..

[150] Tsutsumi Y., Inada K., Morita K., Suzuki T. (1996). T-cell lymphomas diffusely involving the intestine: Report of two rare cases. Jpn. J. Clin. Oncol..

[151] Zivny J., Banner B.F., Agrawal S., Pihan G., Barnard G.F. (2004). CD4+ T-cell lymphoproliferative disorder of the gut clinically mimicking celiac sprue. Dig. Dis. Sci..

[152] Soderquist C.R., Patel N., Murty V.V., Betman S., Aggarwal N., Young K.H., Xerri L., Leeman-Neill R., Lewis S.K., Green P.H. (2020). Genetic and phenotypic characterization of indolent T-cell lymphoproliferative disorders of the gastrointestinal tract. Haematologica.

[153] Leventaki V., Manning J.T., Luthra R., Mehta P., Oki Y., Romaguera J.E., Medeiros L.J., Vega F. (2014). Indolent peripheral T-cell lymphoma involving the gastrointestinal tract. Hum. Pathol..

[154] Malamut G., Meresse B., Kaltenbach S., Derrieux C., Verkarre V., Macintyre E., Ruskone-Fourmestraux A., Fabiani B., Radford-Weiss I., Brousse N. (2014). Small intestinal CD4+ T-cell lymphoma is a heterogenous entity with common pathology features. Clin. Gastroenterol. Hepatol..

[155] Sharma A., Oishi N., Boddicker R.L., Hu G., Benson H.K., Ketterling R.P., Greipp P.T., Knutson D.L., Kloft-Nelson S.M., He R. (2018). Recurrent STAT3-JAK2 fusions in indolent T-cell lymphoproliferative disorder of the gastrointestinal tract. Blood.

[156] Ranheim E.A., Jones C., Zehnder J.L., Warnke R., Yuen A. (2000). Spontaneously relapsing clonal, mucosal cytotoxic T-cell lymphoproliferative disorder: Case report and review of the literature. Am. J. Surg. Pathol..

[157] Egan L.J., Walsh S.V., Stevens F.M., Connolly C.E., Egan E.L., McCarthy C.F. (1995). Celiac-associated lymphoma. A single institution experience of 30 cases in the combination chemotherapy era. J. Clin. Gastroenterol..

[158] Wang X., Ng C.S., Chen C., Yu G., Yin W. (2018). An unusual case report of indolent T-cell lymphoproliferative disorder with aberrant CD20 expression involving the gastrointestinal tract and bone marrow. Diagn. Pathol..

[159] Cheminant M., Bruneau J., Malamut G., Sibon D., Guegan N., van Gils T., Cording S., Trinquand A., Verkarre V., Lhermitte L. (2019). NKp46 is a diagnostic biomarker and may be a therapeutic target in gastrointestinal T-cell lymphoproliferative diseases: A CELAC study. Gut.

[160] Matnani R., Ganapathi K.A., Lewis S.K., Green P.H., Alobeid B., Bhagat G. (2017). Indolent T- and NK-cell lymphoproliferative disorders of the gastrointestinal tract: A review and update. Hematol. Oncol..

[161] Laabi Y., Gras M.P., Carbonnel F., Brouet J.C., Berger R., Larsen C.J., Tsapis A. (1992). A new gene, BCM, on chromosome 16 is fused to the interleukin 2 gene by a t(4;16)(q26;p13) translocation in a malignant T cell lymphoma. EMBO J..

[162] Singh P., Arora A., Strand T.A., Leffler D.A., Catassi C., Green P.H., Kelly C.P., Ahuja V., Makharia G.K. (2018). Global Prevalence of Celiac Disease: Systematic Review and Meta-analysis. Clin. Gastroenterol. Hepatol.

[163] Barker J.M., Liu E. (2008). Celiac disease: Pathophysiology, clinical manifestations, and associated autoimmune conditions. Adv. Pediatr..

[164] Ianiro G., Gasbarrini A., Cammarota G. (2013). Endoscopic tools for the diagnosis and evaluation of celiac disease. World J. Gastroenterol..

[165] Dai Y., Zhang Q., Olofson A.M., Jhala N., Liu X. (2019). Celiac Disease: Updates on Pathology and Differential Diagnosis. Adv. Anat. Pathol..

[166] Dickson B.C., Streutker C.J., Chetty R. (2006). Coeliac disease: An update for pathologists. J. Clin. Pathol..

[167] Sánchez-Castañon M., Castro B.G., Toca M., Santacruz C., Arias-Loste M., Iruzubieta P., Crespo J., López-Hoyos M. (2016). Intraepithelial lymphocytes subsets in different forms of celiac disease. Autoimmun. Highlights.

[168] Steenholt J.V., Nielsen C., Baudewijn L., Staal A., Rasmussen K.S., Sabir H.J., Barington T., Husby S., Toft-Hansen H. (2017). The composition of T cell subtypes in duodenal biopsies are altered in coeliac disease patients. PLoS ONE.

[169] Hüe S., Mention J.J., Monteiro R.C., Zhang S., Cellier C., Schmitz J., Verkarre V., Fodil N., Bahram S., Cerf-Bensussan N. (2004). A direct role for NKG2D/MICA interaction in villous atrophy during celiac disease. Immunity.

[170] Jabri B., De Serre N.P.M., Cellier C., Evans K., Gache C., Carvalho C., Mougenot J.F., Allez M., Jian R., Desreumaux P. (2000). Selective expansion of intraepithelial lymphocytes expressing the HLA-E–specific natural killer receptor CD94 in celiac disease. Gastroenterology.

[171] Hudacko R., Kathy Zhou X., Yantiss R.K. (2013). Immunohistochemical stains for CD3 and CD8 do not improve detection of gluten-sensitive enteropathy in duodenal biopsies. Mod. Pathol..

[172] Biagi F., Corazza G.R. (2001). Defining gluten refractory enteropathy. Eur. J. Gastroenterol. Hepatol..

[173] Al-Toma A., Verbeek W.H., Hadithi M., von Blomberg B.M., Mulder C.J. (2007). Survival in refractory coeliac disease and enteropathy-associated T-cell lymphoma: Retrospective evaluation of single-centre experience. Gut.

[174] Malamut G., Afchain P., Verkarre V., Lecomte T., Amiot A., Damotte D., Bouhnik Y., Colombel J.F., Delchier J.C., Allez M. (2009). Presentation and long-term follow-up of refractory celiac disease: Comparison of type I with type II. Gastroenterology.

[175] Rubio-Tapia A., Kelly D.G., Lahr B.D., Dogan A., Wu T.T., Murray J.A. (2009). Clinical staging and survival in refractory celiac disease: A single center experience. Gastroenterology.

[176] Daum S., Wahnschaffe U., Glasenapp R., Borchert M., Ullrich R., Zeitz M., Faiss S. (2007). Capsule endoscopy in refractory celiac disease. Endoscopy.

[177] Daum S., Weiss D., Hummel M., Ullrich R., Heise W., Stein H., Riecken E.O., Foss H.D., Intestinal Lymphoma Study Group (2001). Frequency of clonal intraepithelial T lymphocyte proliferations in enteropathy-type intestinal T cell lymphoma, coeliac disease, and refractory sprue. Gut.

[178] Cellier C., Delabesse E., Helmer C., Patey N., Matuchansky C., Jabri B., Macintyre E., Cerf-Bensussan N., Brousse N. (2000). Refractory sprue, coeliac disease, and enteropathy-associated T-cell lymphoma. French Coeliac Disease Study Group. Lancet.

[179] Cellier C., Patey N., Mauvieux L., Jabri B., Delabesse E., Cervoni J.P., Burtin M.L., Guy-Grand D., Bouhnik Y., Modigliani R. (1998). Abnormal intestinal intraepithelial lymphocytes in refractory sprue. Gastroenterology.

[180] Tjon J.M., Verbeek W.H., Kooy-Winkelaar Y.M., Nguyen B.H., van der Slik A.R., Thompson A., Heemskerk M.H., Schreurs M.W., Dekking L.H., Mulder C.J. (2008). Defective synthesis or association of T-cell receptor chains underlies loss of surface T-cell receptor-CD3 expression in enteropathy-associated T-cell lymphoma. Blood.

[181] Verbeek W.H., Goerres M.S., von Blomberg B.M., Oudejans J.J., Scholten P.E., Hadithi M., Al-Toma A., Schreurs M.W., Mulder C.J. (2008). Flow cytometric determination of aberrant intra-epithelial lymphocytes predicts T-cell lymphoma development more accurately than T-cell clonality analysis in Refractory Celiac Disease. Clin. Immunol..

[182] Malamut G., Meresse B., Cellier C., Cerf-Bensussan N. (2012). Refractory celiac disease: From bench to bedside. Semin Immunopathol..

[183] van Wanrooij R.L., Muller D.M., Neefjes-Borst E.A., Meijer J., Koudstaal L.G., Heideman D.A., Bontkes H.J., von Blomberg B.M., Bouma G., Mulder C.J. (2014). Optimal strategies to identify aberrant intra-epithelial lymphocytes in refractory coeliac disease. J. Clin. Immunol..

[184] Hussein S., Gindin T., Lagana S.M., Arguelles-Grande C., Krishnareddy S., Alobeid B., Lewis S.K., Mansukhani M.M., Green P.H.R., Bhagat G. (2018). Clonal T cell receptor gene rearrangements in coeliac disease: Implications for diagnosing refractory coeliac disease. J. Clin. Pathol..

[185] Tack G.J., van Wanrooij R.L., Langerak A.W., Tjon J.M., von Blomberg B.M., Heideman D.A., van Bergen J., Koning F., Bouma G., Mulder C.J. (2012). Origin and immunophenotype of aberrant IEL in RCDII patients. Mol. Immunol..

[186] Foss F.M., Horwitz S.M., Civallero M., Bellei M., Marcheselli L., Kim W.S., Cabrera M.E., Dlouhy I., Nagler A., Advani R.H. (2020). Incidence and outcomes of rare T cell lymphomas from the T Cell Project: Hepatosplenic, enteropathy associated and peripheral gamma delta T cell lymphomas. Am. J. Hematol..

[187] Verbeek W., van de water J., Al-Toma A., Oudejans J., Mulder C., Coupe V. (2008). Incidence of enteropathy—Associated T-cell lymphoma: A nation-wide study of a population-based registry in The Netherlands. Scand. J. Gastroenterol..

[188] Sharaiha R.Z., Lebwohl B., Reimers L., Bhagat G., Green P.H., Neugut A.I. (2012). Increasing incidence of enteropathy-associated T-cell lymphoma in the United States, 1973–2008. Cancer.

[189] Yoon S.E., Song Y., Kim S.J., Yoon D.H., Chen T.Y., Koh Y., Kang K.W., Lee H.S., Tay K.K.W., Lim S.T. (2021). Comprehensive analysis of peripheral T-cell and natural killer/T-cell lymphoma in Asian patients: A multinational, multicenter, prospective registry study in Asia. Lancet Reg. Health West. Pac..

[190] Delabie J., Holte H., Vose J.M., Ullrich F., Jaffe E.S., Savage K.J., Connors J.M., Rimsza L., Harris N.L., Muller-Hermelink K. (2011). Enteropathy-associated T-cell lymphoma: Clinical and histological findings from the international peripheral T-cell lymphoma project. Blood.

[191] Bhagat G., Chott A., WHO Classification of Tumours Editorial Board (2019). Enteropathy-associated T-cell lymphoma. Digestive System Tumours.

[192] Jaffe E.S., Chott A., Ott G., Chan J.K.C., Bhagat G., Tan S.Y., Stein H., Isaacson P.G., Swerdlow S.H., Campo E., Harris N.L., Jaffe E.S., Pileri S.A., Stein H., Thiele J. (2017). Intestinal T-cell lymphoma. WHO Classification of Tumours of Haematopoietic and Lymphoid Tissues.

[193] Howell W.M., Leung S.T., Jones D.B., Nakshabendi I., Hall M.A., Lanchbury J.S., Ciclitira P.J., Wright D.H. (1995). HLA-DRB, -DQA, and -DQB polymorphism in celiac disease and enteropathy-associated T-cell lymphoma. Common features and additional risk factors for malignancy. Hum. Immunol..

[194] Malamut G., Chandesris O., Verkarre V., Meresse B., Callens C., Macintyre E., Bouhnik Y., Gornet J.M., Allez M., Jian R. (2013). Enteropathy associated T cell lymphoma in celiac disease: A large retrospective study. Dig. Liver Dis..

[195] Sieniawski M., Angamuthu N., Boyd K., Chasty R., Davies J., Forsyth P., Jack F., Lyons S., Mounter P., Revell P. (2010). Evaluation of enteropathy-associated T-cell lymphoma comparing standard therapies with a novel regimen including autologous stem cell transplantation. Blood.

[196] Isaacson P.G., Du M.Q. (2005). Gastrointestinal lymphoma: Where morphology meets molecular biology. J. Pathol..

[197] Di Sabatino A., Biagi F., Gobbi P.G., Corazza G.R. (2012). How I treat enteropathy-associated T-cell lymphoma. Blood.

[198] Nishimura M.F., Nishimura Y., Nishikori A., Yoshino T., Sato Y. (2021). Primary Gastrointestinal T-Cell Lymphoma and Indolent Lymphoproliferative Disorders: Practical Diagnostic and Treatment Approaches. Cancers.

[199] van Wanrooij R.L., de Jong D., Langerak A.W., Ylstra B., van Essen H.F., Heideman D.A., Bontkes H.J., Mulder C.J., Bouma G. (2015). Novel variant of EATL evolving from mucosal gammadelta-T-cells in a patient with type I RCD. BMJ Open Gastroenterol..

[200] Ting Y.T., Dahal-Koirala S., Kim H.S.K., Qiao S.W., Neumann R.S., Lundin K.E.A., Petersen J., Reid H.H., Sollid L.M., Rossjohn J. (2020). A molecular basis for the T cell response in HLA-DQ2.2 mediated celiac disease. Proc. Natl. Acad. Sci. USA.

[201] Vader W., Stepniak D., Kooy Y., Mearin L., Thompson A., van Rood J.J., Spaenij L., Koning F. (2003). The HLA-DQ2 gene dose effect in celiac disease is directly related to the magnitude and breadth of gluten-specific T cell responses. Proc. Natl. Acad. Sci. USA.

[202] Al-Toma A., Goerres M.S., Meijer J.W., Pena A.S., Crusius J.B., Mulder C.J. (2006). Human leukocyte antigen-DQ2 homozygosity and the development of refractory celiac disease and enteropathy-associated T-cell lymphoma. Clin. Gastroenterol. Hepatol..

[203] Costes L.M.M., Lindenbergh-Kortleve D.J., van Berkel L.A., Veenbergen S., Raatgeep H.R.C., Simons-Oosterhuis Y., van Haaften D.H., Karrich J.J., Escher J.C., Groeneweg M. (2019). IL-10 signaling prevents gluten-dependent intraepithelial CD4(+) cytotoxic T lymphocyte infiltration and epithelial damage in the small intestine. Mucosal Immunol..

[204] Maiuri L., Troncone R., Mayer M., Coletta S., Picarelli A., De Vincenzi M., Pavone V., Auricchio S. (1996). In vitro activities of A-gliadin-related synthetic peptides: Damaging effect on the atrophic coeliac mucosa and activation of mucosal immune response in the treated coeliac mucosa. Scand. J. Gastroenterol..

[205] Meresse B., Chen Z., Ciszewski C., Tretiakova M., Bhagat G., Krausz T.N., Raulet D.H., Lanier L.L., Groh V., Spies T. (2004). Coordinated induction by IL15 of a TCR-independent NKG2D signaling pathway converts CTL into lymphokine-activated killer cells in celiac disease. Immunity.

[206] Di Sabatino A., Ciccocioppo R., Cupelli F., Cinque B., Millimaggi D., Clarkson M.M., Paulli M., Cifone M.G., Corazza G.R. (2006). Epithelium derived interleukin 15 regulates intraepithelial lymphocyte Th1 cytokine production, cytotoxicity, and survival in coeliac disease. Gut.

[207] Zeng R., Spolski R., Finkelstein S.E., Oh S., Kovanen P.E., Hinrichs C.S., Pise-Masison C.A., Radonovich M.F., Brady J.N., Restifo N.P. (2005). Synergy of IL-21 and IL-15 in regulating CD8+ T cell expansion and function. J. Exp. Med..

[208] Mention J.J., Ben Ahmed M., Begue B., Barbe U., Verkarre V., Asnafi V., Colombel J.F., Cugnenc P.H., Ruemmele F.M., McIntyre E. (2003). Interleukin 15: A key to disrupted intraepithelial lymphocyte homeostasis and lymphomagenesis in celiac disease. Gastroenterology.

[209] Sarra M., Cupi M.L., Monteleone I., Franze E., Ronchetti G., Di Sabatino A., Gentileschi P., Franceschilli L., Sileri P., Sica G. (2013). IL-15 positively regulates IL-21 production in celiac disease mucosa. Mucosal Immunol..

[210] Ebert E.C. (2009). Interleukin 21 up-regulates perforin-mediated cytotoxic activity of human intra-epithelial lymphocytes. Immunology.

[211] Parrish-Novak J., Dillon S.R., Nelson A., Hammond A., Sprecher C., Gross J.A., Johnston J., Madden K., Xu W., West J. (2000). Interleukin 21 and its receptor are involved in NK-cell expansion and regulation of lymphocyte function. Nature.

[212] Malamut G., El Machhour R., Montcuquet N., Martin-Lanneree S., Dusanter-Fourt I., Verkarre V., Mention J.J., Rahmi G., Kiyono H., Butz E.A. (2010). IL-15 triggers an antiapoptotic pathway in human intraepithelial lymphocytes that is a potential new target in celiac disease-associated inflammation and lymphomagenesis. J. Clin. Investig..

[213] Chander U., Leeman-Neill R.J., Bhagat G. (2018). Pathogenesis of Enteropathy-Associated T Cell Lymphoma. Curr. Hematol. Malig. Rep..

[214] Deleeuw R.J., Zettl A., Klinker E., Haralambieva E., Trottier M., Chari R., Ge Y., Gascoyne R.D., Chott A., Muller-Hermelink H.K. (2007). Whole-genome analysis and HLA genotyping of enteropathy-type T-cell lymphoma reveals 2 distinct lymphoma subtypes. Gastroenterology.

[215] Ondrejka S.L., Moffitt A.B., Tse E., Hsi E.D., Goodlad J.R., au-Yeung R., Kwong Y.-L., Srivastava G., Gascoyne R.D., Rajagopalan D. (2015). Whole Exome Sequencing of Type 1 and Type 2 Enteropathy-Associated T Cell Lymphoma Reveals Genetic Basis of Eatl Oncogenesis. Blood.

[216] Cording S., Lhermitte L., Malamut G., Berrabah S., Trinquand A., Guegan N., Villarese P., Kaltenbach S., Meresse B., Khater S. (2022). Oncogenetic landscape of lymphomagenesis in coeliac disease. Gut.

[217] Moffitt A.B., Ondrejka S.L., McKinney M., Rempel R.E., Goodlad J.R., Teh C.H., Leppa S., Mannisto S., Kovanen P.E., Tse E. (2017). Enteropathy-associated T cell lymphoma subtypes are characterized by loss of function of SETD2. J. Exp. Med..

[218] Takeshita M., Nakamura S., Kikuma K., Nakayama Y., Nimura S., Yao T., Urabe S., Ogawara S., Yonemasu H., Matsushita Y. (2011). Pathological and immunohistological findings and genetic aberrations of intestinal enteropathy-associated T cell lymphoma in Japan. Histopathology.

[219] Ko Y.H., Karnan S., Kim K.M., Park C.K., Kang E.S., Kim Y.H., Kang W.K., Kim S.J., Kim W.S., Lee W.Y. (2010). Enteropathy-associated T-cell lymphoma--a clinicopathologic and array comparative genomic hybridization study. Hum. Pathol..

[220] Chan J.K., Chan A.C., Cheuk W., Wan S.K., Lee W.K., Lui Y.H., Chan W.K. (2011). Type II enteropathy-associated T-cell lymphoma: A distinct aggressive lymphoma with frequent gammadelta T-cell receptor expression. Am. J. Surg. Pathol..

[221] Bhagat G., Tan S.Y., WHO Classification of Tumours Editorial Board (2019). Intestinal T-cell lymphoma NOS. Digestive System Tumours.

[222] Tan S.Y., de Leval L., WHO Classification of Tumours Editorial Board (2019). Monomorphic epitheliotropic intestinal T-cell lymphoma. Digestive System Tumours.

[223] Cavalieri D., Tournilhac O., Missiglia E., Bonnet C., Ledoux-Pilon A., Bisig B., Cairoli A., Poullot E., Fataccioli V., Parrens M. (2021). Momomorphic epitheliotropic intestinal T-cell lymphoma (MEITL): Clinico-pathological analysis of a multicenter European cohort. Hematol. Oncol..

[224] Haddad P.A., Dadi N. (2020). Clinicopathologic Determinants of Survival in Monomorphic Epitheliotropic Intestinal T-Cell Lymphoma (MEITL): Analysis of a Pooled Database. Blood.

[225] Tan S.Y., Chuang S.S., Tang T., Tan L., Ko Y.H., Chuah K.L., Ng S.B., Chng W.J., Gatter K., Loong F. (2013). Type II EATL (epitheliotropic intestinal T-cell lymphoma): A neoplasm of intra-epithelial T-cells with predominant CD8αα phenotype. Leukemia.

[226] Tse E., Gill H., Loong F., Kim S.J., Ng S.B., Tang T., Ko Y.H., Chng W.J., Lim S.T., Kim W.S. (2012). Type II enteropathy-associated T-cell lymphoma: A multicenter analysis from the Asia Lymphoma Study Group. Am. J. Hematol..

[227] Garcia-Herrera A., Song J.Y., Chuang S.S., Villamor N., Colomo L., Pittaluga S., Alvaro T., Rozman M., de Anda Gonzalez J., Arrunategui A.M. (2011). Nonhepatosplenic gammadelta T-cell lymphomas represent a spectrum of aggressive cytotoxic T-cell lymphomas with a mainly extranodal presentation. Am. J. Surg. Pathol..

[228] Ishibashi H., Nimura S., Kayashima Y., Takamatsu Y., Aoyagi K., Harada N., Kadowaki M., Kamio T., Sakisaka S., Takeshita M. (2016). Multiple lesions of gastrointestinal tract invasion by monomorphic epitheliotropic intestinal T-cell lymphoma, accompanied by duodenal and intestinal enteropathy-like lesions and microscopic lymphocytic proctocolitis: A case series. Diagn. Pathol..

[229] Lu S., Zhou G., Chen M., Liu W., Zhao S. (2021). Monomorphic Epitheliotropic Intestinal T-cell Lymphoma of the Stomach: Two Case Reports and a Literature Review. Int. J. Surg. Pathol..

[230] Antoniadou F., Dimitrakopoulou A., Voutsinas P.M., Vrettou K., Vlahadami I., Voulgarelis M., Korkolopoulou P., Kafasi N., Mikou P. (2017). Monomorphic epitheliotropic intestinal T-cell lymphoma in pleural effusion: A case report. Diagn. Cytopathol..

[231] Chan T.S.Y., Lee E., Khong P.-L., Tse E.W.C., Kwong Y.-L. (2018). Positron emission tomography computed tomography features of monomorphic epitheliotropic intestinal T-cell lymphoma. Hematology.

[232] Chen Y., Tan S.-Y., Petersson B.F., Khor Y.M., Gopalakrishnan S.K., Tan D. (2017). Occult recurrence of monomorphic epitheliotropic intestinal T-cell lymphoma and the role of MATK gene expression in diagnosis. Hematol. Oncol..

[233] Morimoto A., Fujioka Y., Ushiku T., Kurokawa M. (2021). Monomorphic Epitheliotropic Intestinal T-cell Lymphoma Invades Brain. Intern. Med..

[234] Ritter J.P., Flores R., Nazarullah A. (2020). Monomorphic Epitheliotropic Intestinal T-Cell Lymphoma (MEITL) Presenting As An Obstructive Pancreaticoduodenal Mass: A Case Report. Am. J. Clin. Pathol..

[235] Suzuki Y., Minemura H., Tomita H., Saito M., Watanabe N., Umeda T., Kawamata T., Rikimaru M., Morimoto J., Koizumi T. (2020). Monomorphic Epitheliotropic Intestinal T-cell Lymphoma Involving the Lung and Brain: A Rare Case Study. Intern. Med..

[236] Aoki Y., Sujino T., Takabayashi K., Mutakuchi M., Emoto K., Hosoe N., Ogata H., Kanai T. (2021). Various Endoscopic Features in Monomorphic Epitheliotropic Intestinal T-Cell Lymphoma. Case Rep. Gastroenterol..

[237] Hong Y.S., Woo Y.S., Park G., Lee K., Kang S.H., Lee H.W., Kim E.R., Hong S.N., Chang D.K., Kim Y.H. (2016). Endoscopic Findings of Enteropathy-Associated T-Cell Lymphoma Type II: A Case Series. Gut Liver.

[238] Ishibashi H., Nimura S., Hirai F., Harada N., Iwasaki H., Kawauchi S., Oshiro Y., Matsuyama A., Nakamura S., Takamatsu Y. (2020). Endoscopic and clinicopathological characteristics of colorectal T/NK-cell lymphoma. Diagn. Pathol..

[239] Tian S., Xiao S.Y., Chen Q., Liu H., Ping J. (2019). Monomorphic epitheliotropic intestinal T-cell lymphoma may mimic intestinal inflammatory disorders. Int. J. Immunopathol. Pharm..

[240] Kitano Y., Oura S., Mushiake Y., Makimoto S. (2021). Monomorphic Epitheliotropic Intestinal T-Cell Lymphoma in the Ileum with Successful Preoperative Endoscopic Evaluation. Case Rep. Oncol..

[241] Wilson A.L., Swerdlow S.H., Przybylski G.K., Surti U., Choi J.K., Campo E., Trucco M.M., Van Oss S.B., Felgar R.E. (2013). Intestinal γδ T-cell lymphomas are most frequently of type II enteropathy-associated T-cell type. Hum. Pathol..

[242] Mutzbauer G., Maurus K., Buszello C., Pischimarov J., Roth S., Rosenwald A., Chott A., Geissinger E. (2018). SYK expression in monomorphic epitheliotropic intestinal T-cell lymphoma. Mod. Pathol..

[243] Tomita S., Kikuti Y.Y., Carreras J., Kojima M., Ando K., Takasaki H., Sakai R., Takata K., Yoshino T., Bea S. (2015). Genomic and immunohistochemical profiles of enteropathy-associated T-cell lymphoma in Japan. Mod. Pathol..

[244] Tomita S., Kikuti Y.Y., Carreras J., Nakamura N. (2019). Monomorphic epitheliotropic intestinal T-cell lymphoma with T-cell receptor (TCR) of silent phenotype shows rearrangement of TCRβ or TCRγ gene. Pathol. Int..

[245] Chott A., Haedicke W., Mosberger I., Fodinger M., Winkler K., Mannhalter C., Muller-Hermelink H.K. (1998). Most CD56+ intestinal lymphomas are CD8+CD5-T-cell lymphomas of monomorphic small to medium size histology. Am. J. Pathol..

[246] Tan S.Y., Ooi A.S., Ang M.K., Koh M., Wong J.C., Dykema K., Ngeow J., Loong S., Gatter K., Tan L. (2011). Nuclear expression of MATK is a novel marker of type II enteropathy-associated T-cell lymphoma. Leukemia.

[247] Nairismagi M.L., Tan J., Lim J.Q., Nagarajan S., Ng C.C., Rajasegaran V., Huang D., Lim W.K., Laurensia Y., Wijaya G.C. (2016). JAK-STAT and G-protein-coupled receptor signaling pathways are frequently altered in epitheliotropic intestinal T-cell lymphoma. Leukemia.

[248] Okumura K., Ikebe M., Shimokama T., Takeshita M., Kinjo N., Sugimachi K., Higashi H. (2012). An unusual enteropathy-associated T-cell lymphoma with MYC translocation arising in a Japanese patient: A case report. World J. Gastroenterol..

[249] Chen C., Gong Y., Yang Y., Xia Q., Rao Q., Shao Y., Zhu L., Zhang J., Li X., Ji P. (2021). Clinicopathological and molecular genomic features of monomorphic epitheliotropic intestinal T-cell lymphoma in the Chinese population: A study of 20 cases. Diagn. Pathol..

[250] Roberti A., Dobay M.P., Bisig B., Vallois D., Boechat C., Lanitis E., Bouchindhomme B., Parrens M.C., Bossard C., Quintanilla-Martinez L. (2016). Type II enteropathy-associated T-cell lymphoma features a unique genomic profile with highly recurrent SETD2 alterations. Nat. Commun..

[251] Tomita S., Kikuti Y.Y., Carreras J., Sakai R., Takata K., Yoshino T., Bea S., Campo E., Missiaglia E., Bouilly J. (2020). Monomorphic Epitheliotropic Intestinal T-Cell Lymphoma in Asia Frequently Shows SETD2 Alterations. Cancers.

[252] Clarke L., Adduri R.S., Smyth P., Quinn F., Jeffers M., Dunne B., O’Leary J., McKiernan S., Vandenberghe E., Pyne S. (2018). Potentially important miRNAs in enteropathy-associated T-cell lymphoma pathogenesis: A pilot study. Leuk. Res. Rep..

[253] Park S., Ko Y.H. (2014). Peripheral T cell lymphoma in Asia. Int. J. Hematol..

[254] Ciccocioppo R., Croci G.A., Biagi F., Vanoli A., Alvisi C., Cavenaghi G., Riboni R., Arra M., Gobbi P.G., Paulli M. (2018). Intestinal T-cell lymphoma with enteropathy-associated T-cell lymphoma-like features arising in the setting of adult autoimmune enteropathy. Hematol. Oncol..

[255] Attygalle A.D., Cabecadas J., Gaulard P., Jaffe E.S., de Jong D., Ko Y.H., Said J., Klapper W. (2014). Peripheral T-cell and NK-cell lymphomas and their mimics; taking a step forward—report on the lymphoma workshop of the XVIth meeting of the European Association for Haematopathology and the Society for Hematopathology. Histopathology.

[256] Kawamoto K., Nakamura S., Iwashita A., Watanabe J., Oshiro Y., Nakayama Y., Nimura S., Kimura N., Aoyagi K., Yao T. (2009). Clinicopathological characteristics of primary gastric T-cell lymphoma. Histopathology.

[257] Sun J., Lu Z., Yang D., Chen J. (2011). Primary intestinal T-cell and NK-cell lymphomas: A clinicopathological and molecular study from China focused on type II enteropathy-associated T-cell lymphoma and primary intestinal NK-cell lymphoma. Mod. Pathol..

[258] Nicolae A., Xi L., Pham T.H., Pham T.A., Navarro W., Meeker H.G., Pittaluga S., Jaffe E.S., Raffeld M. (2016). Mutations in the JAK/STAT and RAS signaling pathways are common in intestinal T-cell lymphomas. Leukemia.

[259] Kwong Y.-L., Kim W.S., Lim S.T., Kim S.J., Tang T., Tse E., Leung A.Y.H., Chim C.-S. (2012). SMILE for natural killer/T-cell lymphoma: Analysis of safety and efficacy from the Asia Lymphoma Study Group. Blood.

[260] Allen P.B., Lechowicz M.J. (2019). Management of NK/T-Cell Lymphoma, Nasal Type. J. Oncol. Pract..

[261] Tse E., Kwong Y.-L. (2013). How I treat NK/T-cell lymphomas. Blood.

[262] Lee J., Kim W.S., Park Y.H., Park S.H., Park K.W., Kang J.H., Lee S.S., Lee S.I., Lee S.H., Kim K. (2005). Nasal-type NK/T cell lymphoma: Clinical features and treatment outcome. Br. J. Cancer.

[263] Yamaguchi M., Kwong Y.-L., Kim W.S., Maeda Y., Hashimoto C., Suh C., Izutsu K., Ishida F., Isobe Y., Sueoka E. (2011). Phase II Study of SMILE Chemotherapy for Newly Diagnosed Stage IV, Relapsed, or Refractory Extranodal Natural Killer (NK)/T-Cell Lymphoma, Nasal Type: The NK-Cell Tumor Study Group Study. J. Clin. Oncol..

[264] Lee J., Au W.Y., Park M.J., Suzumiya J., Nakamura S., Kameoka J., Sakai C., Oshimi K., Kwong Y.L., Liang R. (2008). Autologous hematopoietic stem cell transplantation in extranodal natural killer/T cell lymphoma: A multinational, multicenter, matched controlled study. Biol. Blood Marrow Transplant..

[265] Li X., Cui Y., Sun Z., Zhang L., Li L., Wang X., Wu J., Fu X., Ma W., Zhang X. (2016). DDGP versus SMILE in Newly Diagnosed Advanced Natural Killer/T-Cell Lymphoma: A Randomized Controlled, Multicenter, Open-label Study in China. Clin. Cancer Res..

[266] Wang J.H., Wang L., Liu C.C., Xia Z.J., Huang H.Q., Lin T.Y., Jiang W.Q., Lu Y. (2016). Efficacy of combined gemcitabine, oxaliplatin and pegaspargase (P-gemox regimen) in patients with newly diagnosed advanced-stage or relapsed/refractory extranodal NK/T-cell lymphoma. Oncotarget.

[267] Zhang L., Jia S., Ma Y., Li L., Li X., Wang X., Fu X., Ma W., Qin Y., Li W. (2016). Efficacy and safety of cisplatin, dexamethasone, gemcitabine and pegaspargase (DDGP) regimen in newly diagnosed, advanced-stage extranodal natural killer/T-cell lymphoma: Interim analysis of a phase 4 study NCT01501149. Oncotarget.

[268] Zhao Q., Fan S., Chang Y., Liu X., Li W., Ma Q., Li Y., Wang Y., Zhang L., Zhang M. (2019). Clinical efficacy of cisplatin, dexamethasone, gemcitabine and pegaspargase (DDGP) in the initial treatment of advanced stage (stage III-IV) extranodal NK/T-cell lymphoma, and its correlation with Epstein-Barr virus. Cancer Manag. Res..

[269] Hu B., Oki Y. (2018). Novel Immunotherapy Options for Extranodal NK/T-Cell Lymphoma. Front. Oncol..

[270] Lv K., Li X., Yu H., Chen X., Zhang M., Wu X. (2020). Selection of new immunotherapy targets for NK/T cell lymphoma. Am. J. Transl Res..

[271] Aeppli S., Driessen C., Graf L., Hitz F. (2018). Systemic treatment of a patient with relapsed and refractory extranodal NK/T-cell lymphoma (ENKL) and meningeosis leukemica with daratumumab. Hematol. Oncol..

[272] Hari P., Raj R.V., Olteanu H. (2016). Targeting CD38 in Refractory Extranodal Natural Killer Cell-T-Cell Lymphoma. N. Engl. J. Med..

[273] Kim H.K., Moon S.M., Moon J.H., Park J.E., Byeon S., Kim W.S. (2015). Complete remission in CD30-positive refractory extranodal NK/T-cell lymphoma with brentuximab vedotin. Blood Res..

[274] Park S., Kim S.J., Hong J.Y., Yoon D.H., Kim J.S., Kang H.J., Eom H.-S., Lee M.H., Suh C., Kim W.S. (2017). A Phase II Study of Brentuximab Vedotin for Relapsed or Refractory CD30-Positive Non-Hodgkin Lymphomas Other Than Anaplastic Large Cell Lymphoma. Blood.

[275] Poon L.M., Kwong Y.L. (2016). Complete remission of refractory disseminated NK/T cell lymphoma with brentuximab vedotin and bendamustine. Ann. Hematol..

[276] Huang H., Zhu J., Yao M., Kim T.M., Yoon D.H., Cho S.G., Eom H.S., Lim S.T., Yeh S.P., Song Y. (2021). Daratumumab monotherapy for patients with relapsed or refractory natural killer/T-cell lymphoma, nasal type: An open-label, single-arm, multicenter, phase 2 study. J. Hematol. Oncol..

[277] Chan T.S.Y., Li J., Loong F., Khong P.L., Tse E., Kwong Y.L. (2018). PD1 blockade with low-dose nivolumab in NK/T cell lymphoma failing L-asparaginase: Efficacy and safety. Ann. Hematol..

[278] Kwong Y.L., Chan T.S.Y., Tan D., Kim S.J., Poon L.M., Mow B., Khong P.L., Loong F., Au-Yeung R., Iqbal J. (2017). PD1 blockade with pembrolizumab is highly effective in relapsed or refractory NK/T-cell lymphoma failing l-asparaginase. Blood.

[279] Li X., Cheng Y., Zhang M., Yan J., Li L., Fu X., Zhang X., Chang Y., Sun Z., Yu H. (2018). Activity of pembrolizumab in relapsed/refractory NK/T-cell lymphoma. J. Hematol. Oncol..

[280] Kim S.J., Hyeon J., Cho I., Ko Y.H., Kim W.S. (2019). Comparison of Efficacy of Pembrolizumab between Epstein-Barr Virus-Positive and -Negative Relapsed or Refractory Non-Hodgkin Lymphomas. Cancer Res. Treat..

[281] Cai J., Liu P., Huang H., Li Y., Ma S., Zhou H., Tian X., Zhang Y., Gao Y., Xia Y. (2020). Combination of anti-PD-1 antibody with P-GEMOX as a potentially effective immunochemotherapy for advanced natural killer/T cell lymphoma. Signal Transduct. Target..

[282] Farid M., Yau Y.W., Tay K., Quek R., Tao M., Koo G.C., Loong S., Lim S.T. (2011). A promising new regimen for the treatment of advanced extranodal NK/T cell lymphoma. Acta Oncol..

[283] Chen C., He H. (2017). Treatment of relapsed extranodal natural killer/T-cell lymphoma with bortezomib plus fludarabine. Mol. Clin. Oncol..

[284] Moskowitz A.J., Ghione P., Jacobsen E., Ruan J., Schatz J.H., Noor S., Myskowski P., Vardhana S., Ganesan N., Hancock H. (2021). A phase 2 biomarker-driven study of ruxolitinib demonstrates effectiveness of JAK/STAT targeting in T-cell lymphomas. Blood.

[285] Sieniawski M.K., Lennard A.L. (2011). Enteropathy-associated T-cell lymphoma: Epidemiology, clinical features, and current treatment strategies. Curr. Hematol. Malig. Rep..

[286] Sibon D., Khater S., Bruneau J., Brouzes C., Lhermitte L., Molina T.J., Cartron G., Morel V., Malamut G., Chauchet A. (2021). The Eatl-001 Trial: Results of a Phase 2 Study of Brentuximab Vedotin and CHP Followed By Consolidation with High-Dose Therapy—Autologous Stem-Cell Transplantation (HDT-ASCT) in the Frontline Treatment of Patients with Enteropathy-Associated T-Cell Lymphoma. Blood.

[287] Gentille C., Qin Q., Barbieri A., Ravi P.S., Iyer S. (2017). Use of PEG-asparaginase in monomorphic epitheliotropic intestinal T-cell lymphoma, a disease with diagnostic and therapeutic challenges. Ecancermedicalscience.

[288] Vose J., Armitage J., Weisenburger D., International T.C.L.P. (2008). International peripheral T-cell and natural killer/T-cell lymphoma study: Pathology findings and clinical outcomes. J. Clin. Oncol..

[289] Ellin F., Landström J., Jerkeman M., Relander T. (2014). Real-world data on prognostic factors and treatment in peripheral T-cell lymphomas: A study from the Swedish Lymphoma Registry. Blood.

[290] Schmitz N., Trümper L., Ziepert M., Nickelsen M., Ho A.D., Metzner B., Peter N., Loeffler M., Rosenwald A., Pfreundschuh M. (2010). Treatment and prognosis of mature T-cell and NK-cell lymphoma: An analysis of patients with T-cell lymphoma treated in studies of the German High-Grade Non-Hodgkin Lymphoma Study Group. Blood.

[291] Altmann B., Wulf G., Truemper L., d’Amore F., Relander T., Toldbod H., Delabie J.M.A., Rosenwald A., Ziepert M., Loeffler M. (2018). Alemtuzumab Added to CHOP for Treatment of Peripheral T-Cell Lymphoma (PTCL) in Previously Untreated Young and Elderly Patients: Pooled Analysis of the International ACT-1/2 Phase III Trials. Blood.

[292] Gallamini A., Zaja F., Patti C., Billio A., Specchia M.R., Tucci A., Levis A., Manna A., Secondo V., Rigacci L. (2007). Alemtuzumab (Campath-1H) and CHOP chemotherapy as first-line treatment of peripheral T-cell lymphoma: Results of a GITIL (Gruppo Italiano Terapie Innovative nei Linfomi) prospective multicenter trial. Blood.

[293] Wulf G.G., Altmann B., Ziepert M., D’Amore F., Held G., Greil R., Tournilhac O., Relander T., Viardot A., Wilhelm M. (2021). Alemtuzumab plus CHOP versus CHOP in elderly patients with peripheral T-cell lymphoma: The DSHNHL2006-1B/ACT-2 trial. Leukemia.

[294] Foss F.M., Sjak-Shie N.N., Goy A., Advani R., Jacobsen E.D. (2010). Phase II study of denileukin diftitox with CHOP chemotherapy in newly-diagnosed PTCL: CONCEPT trial. J. Clin. Oncol..

[295] Kim S.J., Yoon D.H., Kang H.J., Kim J.S., Park S.K., Kim H.J., Lee J., Ryoo B.Y., Ko Y.H., Huh J. (2012). Bortezomib in combination with CHOP as first-line treatment for patients with stage III/IV peripheral T-cell lymphomas: A multicentre, single-arm, phase 2 trial. Eur. J. Cancer.

[296] Yang H., Li C. (2021). The Combination of Bortezomib with Cyclophosphamide, Epirubicin, Etoposide and Prednisone (BCHEP) Regimen As First-Line Treatment for Untreated Patients with Peripheral T Cell Lymphoma: A Prospective, Single Arm, Phase II Study. Blood.

[297] Escalón M.P., Liu N.S., Yang Y., Hess M., Walker P.L., Smith T.L., Dang N.H. (2005). Prognostic factors and treatment of patients with T-cell non-Hodgkin lymphoma: The M. D. Anderson Cancer Center experience. Cancer.

[298] Gisselbrecht C., Gaulard P., Lepage E., Coiffier B., Brière J., Haioun C., Cazals-Hatem D., Bosly A., Xerri L., Tilly H. (1998). Prognostic significance of T-cell phenotype in aggressive non-Hodgkin’s lymphomas. Groupe d’Etudes des Lymphomes de l’Adulte (GELA). Blood.

[299] Xu Y., Wu X.J., Wang Y., Jin Z.M., Sun A.N., Wu D.P. (2012). Hyper-CVAD chemotherapy or autologous stem cell transplantation in patients with peripheral T cell lymphomas: A single centre report. Chin. Med. J..

[300] Corazzelli G., Frigeri F., Marcacci G., Becchimanzi C., Capobianco G., Arcamone M., Morelli E., Volzone F., Russo F., Pinto A. (2010). Gemcitabine, Ifosfamide, Oxaliplatin (GIFOX) as First-Line Treatment In High-Risk Peripheral T-Cell/NK Lymphomas: A Phase II Trial. Blood.

[301] Dong M., He X.H., Liu P., Qin Y., Yang J.L., Zhou S.Y., Yang S., Zhang C.G., Gui L., Zhou L.Q. (2013). Gemcitabine-based combination regimen in patients with peripheral T-cell lymphoma. Med. Oncol..

[302] Evens A.M., Rosen S.T., Helenowski I., Kline J., Larsen A., Colvin J., Winter J.N., van Besien K.M., Gordon L.I., Smith S.M. (2013). A phase I/II trial of bortezomib combined concurrently with gemcitabine for relapsed or refractory DLBCL and peripheral T-cell lymphomas. Br. J. Haematol..

[303] Yhim H.Y., Kim T., Kim S.J., Shin H.J., Koh Y., Kim J.S., Park J., Park G.S., Kim W.S., Moon J.H. (2021). Combination treatment of copanlisib and gemcitabine in relapsed/refractory PTCL (COSMOS): An open-label phase I/II trial. Ann. Oncol..

[304] Zinzani P.L., Venturini F., Stefoni V., Fina M., Pellegrini C., Derenzini E., Gandolfi L., Broccoli A., Argnani L., Quirini F. (2010). Gemcitabine as single agent in pretreated T-cell lymphoma patients: Evaluation of the long-term outcome. Ann. Oncol..

[305] Hong J.Y., Yoon D.H., Yoon S.E., Kim S.J., Lee H.S., Eom H.-S., Lee H.W., Shin D.-Y., Koh Y., Yoon S.-S. (2019). Pralatrexate in patients with recurrent or refractory peripheral T-cell lymphomas: A multicenter retrospective analysis. Sci. Rep..

[306] O’Connor O.A., Pro B., Pinter-Brown L., Bartlett N., Popplewell L., Coiffier B., Lechowicz M.J., Savage K.J., Shustov A.R., Gisselbrecht C. (2011). Pralatrexate in patients with relapsed or refractory peripheral T-cell lymphoma: Results from the pivotal PROPEL study. J. Clin. Oncol..

[307] Coiffier B., Pro B., Prince H.M., Foss F., Sokol L., Greenwood M., Caballero D., Borchmann P., Morschhauser F., Wilhelm M. (2012). Results from a pivotal, open-label, phase II study of romidepsin in relapsed or refractory peripheral T-cell lymphoma after prior systemic therapy. J. Clin. Oncol..

[308] Coiffier B., Pro B., Prince H.M., Foss F., Sokol L., Greenwood M., Caballero D., Morschhauser F., Wilhelm M., Pinter-Brown L. (2014). Romidepsin for the treatment of relapsed/refractory peripheral T-cell lymphoma: Pivotal study update demonstrates durable responses. J. Hematol. Oncol..

[309] Piekarz R.L., Frye R., Prince H.M., Kirschbaum M.H., Zain J., Allen S.L., Jaffe E.S., Ling A., Turner M., Peer C.J. (2011). Phase 2 trial of romidepsin in patients with peripheral T-cell lymphoma. Blood.

[310] Morschhauser F., Fitoussi O., Haioun C., Thieblemont C., Quach H., Delarue R., Glaisner S., Gabarre J., Bosly A., Lister J. (2013). A phase 2, multicentre, single-arm, open-label study to evaluate the safety and efficacy of single-agent lenalidomide (Revlimid) in subjects with relapsed or refractory peripheral T-cell non-Hodgkin lymphoma: The EXPECT trial. Eur. J. Cancer.

[311] Ruan J., Zain J.M., Palmer B., Jovanovic B., Mi X., Swaroop A., Winter J., Gordon L.I., Karmali R., Pro B. (2021). Multicenter phase II study of romidepsin plus lenalidomide for patients with previously untreated peripheral T-cell lymphoma (PTCL). J. Clin. Oncol..

[312] Toumishey E., Prasad A., Dueck G., Chua N., Finch D., Johnston J., van der Jagt R., Stewart D., White D., Belch A. (2015). Final report of a phase 2 clinical trial of lenalidomide monotherapy for patients with T-cell lymphoma. Cancer.

[313] Umakanthan J.M., Iqbal J., Batlevi C.L., Bouska A., Smith L.M., Shostrom V., Nutsch H., William B.M., Gregory Bociek R., Lunning M. (2019). Phase I/II study of dasatinib and exploratory genomic analysis in relapsed or refractory non-Hodgkin lymphoma. Br. J. Haematol..

[314] William B.M., Hohenstein M., Loberiza F.R., Caponetti G.C., Bociek R.G., Bierman P., Armitage J.O., Chan W.-C., Vose J.M. (2010). Phase I/II Study of Dasatinib In Relapsed or Refractory Non-Hodgkin’s Lymphoma (NHL). Blood.

[315] Barr P.M., Li H., Spier C., Mahadevan D., LeBlanc M., Ul Haq M., Huber B.D., Flowers C.R., Wagner-Johnston N.D., Horwitz S.M. (2015). Phase II Intergroup Trial of Alisertib in Relapsed and Refractory Peripheral T-Cell Lymphoma and Transformed Mycosis Fungoides: SWOG 1108. J. Clin. Oncol..

[316] Friedberg J.W., Mahadevan D., Cebula E., Persky D., Lossos I., Agarwal A.B., Jung J., Burack R., Zhou X., Leonard E.J. (2014). Phase II study of alisertib, a selective Aurora A kinase inhibitor, in relapsed and refractory aggressive B- and T-cell non-Hodgkin lymphomas. J. Clin. Oncol..

[317] O’Connor O.A., Özcan M., Jacobsen E.D., Roncero J.M., Trotman J., Demeter J., Masszi T., Pereira J., Ramchandren R., Beaven A. (2019). Randomized Phase III Study of Alisertib or Investigator’s Choice (Selected Single Agent) in Patients With Relapsed or Refractory Peripheral T-Cell Lymphoma. J. Clin. Oncol..

[318] Quéméner A., Maillasson M., Arzel L., Sicard B., Vomiandry R., Mortier E., Dubreuil D., Jacques Y., Lebreton J., Mathé-Allainmat M. (2017). Discovery of a Small-Molecule Inhibitor of Interleukin 15: Pharmacophore-Based Virtual Screening and Hit Optimization. J. Med. Chem..

